# Evolutionary, structural and functional relationships revealed by comparative analysis of syntenic genes in Rhizobiales

**DOI:** 10.1186/1471-2148-5-55

**Published:** 2005-10-17

**Authors:** Gabriela Guerrero, Humberto Peralta, Alejandro Aguilar, Rafael Díaz, Miguel Angel Villalobos, Arturo Medrano-Soto, Jaime Mora

**Affiliations:** 1 Program of Functional Genomics of Prokaryotes, Centro de Ciencias Genómicas, Universidad Nacional Autónoma de México. Ave. Universidad s/n (P.O. Box 565-A), Cuernavaca, Morelos, 62210, México; 2 Program of Computational Genomics, Centro de Ciencias Genómicas, Universidad Nacional Autónoma de México. Ave. Universidad s/n (P.O. Box 565-A), Cuernavaca, Morelos, 62210, México

## Abstract

**Background:**

Comparative genomics has provided valuable insights into the nature of gene sequence variation and chromosomal organization of closely related bacterial species. However, questions about the biological significance of gene order conservation, or synteny, remain open. Moreover, few comprehensive studies have been reported for rhizobial genomes.

**Results:**

We analyzed the genomic sequences of four fast growing Rhizobiales (*Sinorhizobium meliloti*, *Agrobacterium tumefaciens*, *Mesorhizobium loti *and *Brucella melitensis*). We made a comprehensive gene classification to define chromosomal orthologs, genes with homologs in other replicons such as plasmids, and those which were species-specific. About two thousand genes were predicted to be orthologs in each chromosome and about 80% of these were syntenic. A striking gene colinearity was found in pairs of organisms and a large fraction of the microsyntenic regions and operons were similar. Syntenic products showed higher identity levels than non-syntenic ones, suggesting a resistance to sequence variation due to functional constraints; also, an unusually high fraction of syntenic products contained membranal segments. Syntenic genes encode a high proportion of essential cell functions, presented a high level of functional relationships and a very low horizontal gene transfer rate. The sequence variability of the proteins can be considered the species signature in response to specific niche adaptation. Comparatively, an analysis with genomes of Enterobacteriales showed a different gene organization but gave similar results in the synteny conservation, essential role of syntenic genes and higher functional linkage among the genes of the microsyntenic regions.

**Conclusion:**

Syntenic bacterial genes represent a commonly evolved group. They not only reveal the core chromosomal segments present in the last common ancestor and determine the metabolic characteristics shared by these microorganisms, but also show resistance to sequence variation and rearrangement, possibly due to their essential character. In Rhizobiales and Enterobacteriales, syntenic genes encode a high proportion of essential cell functions and presented a high level of functional relationships.

## Background

A huge amount of information has been obtained from sequencing projects. More than two hundred complete microbial genomes are available to date in public databases and sequencing of a similar number is in progress [1]. Many questions remain unsolved. For example, what is the biological meaning, if any, of gene arrangement in the bacterial chromosome?

Changes in gene sequence and chromosomal rearrangements constitute the main sources of genomic variability. Nonsynonymous substitutions in the first or second nucleotides of the codon change the encoded residue and are thus a driving force of natural selection. Genomic studies in bacteria regarding synonymous and nonsynonymous substitution rates have been published elsewhere [2,3]. Chromosomes show constraints on rearrangement and works dealing with that aspect were recently reviewed by Rocha [4]; he suggested that there is a balance between conservation and change in the organization of the chromosome.

The operon represents the first level of the gene organization. Neighboring genes, especially those in co-directional and in divergent orientation, represent a second organization level because they show a certain functional association revealed by genomic context analysis [5]. Regarding comparisons among closely related species, the gene order conservation, or synteny, represents a third level of organization. Synteny depends on shared ancestry and inter- and intrachromosomal exchanges, and represents a higher relationship between taxa. It was suggested that physiologically important gene clusters could be positively selected, and synteny perhaps reveals the functional constraints of these genes [6]. For the detection of synteny it is necessary first to determine the set of orthologous genes in pairs of organisms and recently an inverse method has proven useful for this [7].

Recombination/transposition events can easily disrupt synteny. Species of *Buchnera *and *Corynebacterium *have low levels of chromosomal rearrangements and lack *recA *and *recBCD *orthologs, respectively [8,9], thus suggesting that recombination is an important factor for loss of synteny. Synteny studies have focused in short gene clusters in eukaryotes [10,11] while whole chromosome comparative analysis has been done in bacteria and archaea [12-18]. For example, there is a striking conservation between the chromosomes of *Escherichia coli *and *Salmonella typhimurium *[19], and also between those of *Brucella melitensis, B. suis *and *Mesorhizobium loti *[20]. However, the synteny analyses for alpha proteobacteria such as those reported in the genome sequence determinations of *Brucella melitensis*, *Sinorhizobium meliloti *and *Agrobacterium tumefaciens *are highly schematic [20-22]. A thorough analysis of Rhizobiales genomes would determine if the key set of genes covering the most important metabolic functions in these organisms are syntenic.

Another factor affecting chromosomal rearrangement is horizontal gene transfer (HGT) which occurs in bacteria [23,24], but estimating its impact on genome organization has proved a daunting task for two reasons. First, the reliability of compositional methods to detect HGT events has been questioned [25,26]. Second, phylogenetic methods, albeit more reliable, are not always applicable and can easily be misleading without proper care. The results of a recently published analysis suggest that codon usage compatibility between alien genes and recipient genomes [27] is a prerequisite for successful HGT events. This premise has been supported by other evidence [28,29].

Among the most accepted methods to deduce functional relationships of proteins are phylogenetic profile [30], gene neighboring [31], and the Rossetta stone method [32]. These methods can give additive information about metabolic networks existing in organisms [33]. ProLinks is a program based on these methods with an extensive library of predicted functional interactions from 83 genomes [34]. Von Mering *et al*. [35], also applying these approaches with the STRING program, found global modularities in functional protein networks. A question remains about whether functional linkage differences exist in syntenic and non-syntenic gene clusters.

Rhizobiales is a prokaryotic order belonging to the alpha proteobacteria subdivision; some rhizobial species are intensively studied for their nitrogen-fixing ability when in symbiosis with leguminous plants. The order comprises both plant symbionts and plant and animal pathogens such as *Rhizobium, Agrobacterium *and *Brucella*, respectively. In rhizobia, genes responsible for the symbiotic interaction are commonly found on large plasmids or incorporated in a particular stretch of the chromosome called the symbiotic island [36-38]. The physiological potential of the rhizobial chromosome allows cell survival under different conditions. For example, an *A. tumefaciens *strain containing the symbiotic plasmid from *Rhizobium etli *induced nodules on legume plants [39,40], and conversely, an *S. meliloti *derivative strain with Ri, the rhizogenic induction plasmid, formed root mats on alfalfa plants [41]. Additionally, there are similarities in the parasitic/symbiotic strategies employed by species of the Rhizobiales [20]. Also, it is possible to find diverse life-styles among the members of Enterobacteriales order (gamma proteobacteria): for example, *Buchnera *is an obligate aphid symbiont; *E. coli *and *S. typhimurium *are common gut inhabitants in mammals; and *Shigella flexneri, Yersinia pestis *and *Erwinia carotovora *are pathogens, either for animals or plants [42].

The complete genome sequences of seven species of Rhizobiales were available by 2004, namely *S. meliloti *[21], *Mesorhizobium loti *[43], *Bradyrhizobium japonicum *[44], *A. tumefaciens *[22,45], *B. melitensis *[46], *B. suis *[20] and *Rhodopseudomonas palustris *[47]. Although their genomes show a certain degree of conservation, variability corresponding to their evolutionary divergence points, microbial life styles and ecological niches was also found. Comparative genomics has captured the attention of researchers as a way of achieving a better understanding of the molecular basis underlying phenomena such as symbiosis and pathogenesis.

We classified the genes of several rhizobial species in order to gain a comprehensive insight into chromosomal conservation and genome rearrangement. Conserved genes among these species can reveal phylogenetic relationships, but also show metabolic strategies useful in understanding the niche diversity in which these organisms usually grow. In particular, syntenic/non-syntenic genes among these species were analyzed in terms of their sequence identity/similarity, physical characteristics of the encoded products and functional relationships among them. Additionally, in order to find more general trends, we compared these results with an analysis performed on genomes belonging to the Enterobacteriales.

## Results

### Approach, strategy and outline

Our main objective was to enhance our understanding of the functional meaning of the gene arrangement on the bacterial chromosome, taking as examples some genomes from the Rhizobiales and Enterobacteriales. We consider that gene neighboring is not a random trait and gives an adaptive advantage to the cell because the proteins produced are likely to perform related functions. Our belief is that the coordinated expression of genes, organized on the chromosome either as operons or clusters, permits the correct integration of metabolic functions.

Our approaches were: i) to obtain a comprehensive gene classification, applicable to each of the species analyzed and suitable to make comparisons among them, and ii) to detect specific gene characteristics (if any) of each of the classes. In the first approach we identified orthologs among chromosomes, defined those that were syntenic, those in a different replicon, and those that were species-specific. For the second approach, we analyzed gene/protein sequences for identity, calculated the horizontal gene transfer rate for each class and the predicted molecular weight and isoelectric point of the peptides, and inferred the functional relationship in syntenic or non-conserved chromosomal regions. The results are presented in the following order: 1) Synteny in Rhizobiales (gene classification of Rhizobiales; gene organization and microsyntenic region formation; synteny and insertion sequences, horizontal gene transfer and codon usage), 2) Synteny in Enterobacteriales, 3) Sequence analysis of the chromosomal predicted orthologs, 4) Physical characteristics of the translated products of syntenic genes, and 5) Functional roles and linkage of chromosomal predicted orthologs. *S. meliloti *was taken as reference organism for the comparisons with each *A. tumefaciens, M. loti*, *B. melitensis *and *E. coli*. *E. coli *was used as base to compare with *S. typhimurium*, *E. carotovora *and *S. meliloti*.

#### 1) Synteny in Rhizobiales

##### Gene classification of Rhizobiales

The selection criteria mentioned in **Methods **were applied to the chromosomes of *S. meliloti *(*Sm*), *A. tumefaciens *(*At*), *M. loti *(*Ml*), and *B. melitensis *(*Bm*). As compared to the chromosome of *S. meliloti*, we found that more than 60% of genes were chromosomal predicted orthologs in the *At *circular chromosome (*At*-C) and *Bm *chromosome I (*Bm*-I), one third in *Ml *chromosome and *Bm *chromosome II (*Bm*-II) and one quarter in the *At *linear chromosome (*At*-L); however, the number of chromosomal predicted orthologs in each organism was similar, about two thousand (Table 1). The *Sm *chromosome presents 3341 genes in 3.65 Mb.

**Table 1:** Gene classification of *Rhizobiales*.

	In comparison with the *Sinorhizobium meliloti *chromosome						
	
	*At*-C	*At*-L	*At*	*Ml*	*Bm*-I	*Bm*-II	*Bm*
Genes in chromosome (length in Mb)	2721(2.84)	1833(2.07)	4554(4.92)	6750(7.04)	2059(2.12)	1139(1.18)	3198(3.30)
Chromosomal orthologs (% of chr. genes)	1737(63.8)	478(26.1)	2215(48.6)	2279(33.8)	1310(63.6)	415(36.4)	1725(53.9)
Syntenic genes (% of chr. orthologs)	1480(85.2)	357(74.7)	1837(82.9)	1624(71.3)	1039(79.3)	272(65.5)	1311(76.0)
Nonsyntenic genes (% of chr. orthologs)	257(14.8)	121(25.3)	378(17.1)	655(28.7)	271(20.7)	143(34.5)	414(24.0)
Microsyntenic regions	160	45	205	227	132	41	173
Syntenic genes in regions (% of synt. genes)	1394(94.2)	325(91.0)	1719(93.6)	1428(87.9)	965(92.9)	230(84.6)	1195(91.1)

Plasmidic homologs (% of chr. genes)	159(5.8)	490(26.7)	649(14.2)	924(13.7)	70(3.4)	134(11.8)	204(6.4)
Homologs in rest of Rhizobiales (% of chr. genes)	459(16.9)	523(28.5)	982(21.6)	1724(25.5)	349(16.9)	368(32.3)	717(22.4)
Species specific genes (% of chr. genes)	366(13.4)	342(18.7)	708(15.5)	1823(27.0)	330(16.0)	222(19.5)	552(17.3)

Operons (except homologs in rest of Rhizobiales)	527	336	863	1140	353	192	545
Syntenic operons (% of operons)	282(53.5)	68(20.2)	350(40.6)	259(22.7)	168(47.6)	43(22.4)	211(38.7)
Nonsyntenic operons (% of operons)	7(1.3)	0	7(0.8)	12(1.0)	8(2.3)	3(1.6)	11(2.0)
Plasmidic operons (% of operons)	15(2.8)	47(14.0)	62(7.2)	88(7.7)	1(0.3)	9(4.7)	10(1.8)
Species specific operons (% of operons)	26(4.9)	27(8.0)	53(6.1)	150(13.2)	20(5.7)	14(7.3)	34(6.2)
Mixed operons (% of operons)	197(37.4)	194(57.7)	391(45.3)	631(55.3)	156(44.2)	123(64.1)	279(51.2)

Chr., chromosomal. Synt., syntenic. *At*-C, *A. tumefaciens *circular chromosome. *At*-L, *A. tumefaciens *linear chromosome. *At*, *A. tumefaciens *chromosomes. *Ml*, *M. loti *chromosome. *Bm*-I, *B. melitensis *chromosome I. *Bm*-II, *B. melitensis *chromosome II. *Bm*, *B. melitensis *chromosomes.

We assessed the chromosomal genes with conserved order or synteny (see **Methods**). Syntenic genes represented about 70–80% of chromosomal predicted orthologs (see Table 1). That is, the conserved chromosomal order of these genes seems favored. The remarkable synteny level is highlighted by a group of 1038 common syntenic genes in all these species. Non-syntenic genes represented from 17% to 35% of the chromosomal predicted orthologs in these organisms. Only 98 non-syntenic genes were common in the four Rhizobiales.

**Figure 1:**
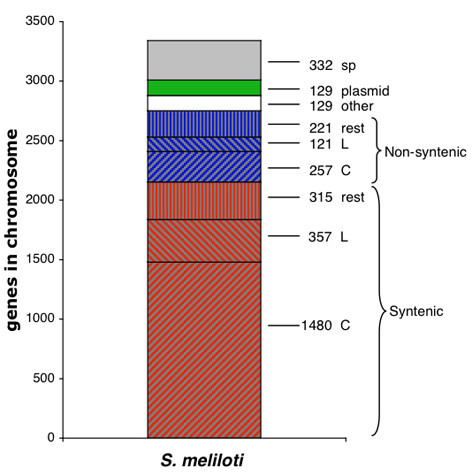
Schematic representation of the *S. meliloti *chromosome (compared with *A. tumefaciens*) according to the classification of predicted orthologs and homologs. Striped bars in red, from the bottom to the top: genes syntenic with the *A. tumefaciens *circular chromosome (denoted with C); syntenic with the *A. tumefaciens *linear chromosome (L); syntenic with the rest of Rhizobiales (rest). Striped bars in blue, from the bottom to the top: genes non-syntenic with the *A. tumefaciens *circular chromosome (C); non-syntenic with *A. tumefaciens *linear chromosome (L); non-syntenic with the rest of Rhizobiales (rest). White bar, homologs in other Rhizobiales chromosomes matched with unidirectional best hits (other). Green bar, homologs in plasmids (plasmid). Gray bar, species-specific genes (sp). Numbers indicate genes in each of the categories.

The remaining categories obtained in this analysis were: homologs present in plasmids, homologs with the rest of rhizobial chromosomes, and those with no orthologs in the public databases (species-specific genes, also known as orphan). Homologs in plasmids were more abundant in the *At*-L,*Ml *and *Bm*-II chromosomes (Table 1). These replicons have special features as commented below and in **Discussion**. Aside from the two organisms being compared, some genes also matched with other rhizobial chromosomes (including *B. japonicum *USDA110) and comprised about a quarter of the chromosomal genes in these species (Table 1). Some of them only matched with unidirectional best hits (homologs). In this context, in an additional analysis between *S. meliloti *and *A. tumefaciens*, we found that 129 genes matched with unidirectional best hit (white fraction, Figure 1), but the rest were predicted orthologs with bidirectional best hits, either syntenic or non-syntenic, and are represented in the red and blue striped bars (denoted as rest, Figure 1), respectively. Species-specific genes were especially abundant in the *M. loti *chromosome, while in the other replicons this class covered at most 20%. One half of these species-specific genes were not present in the COG database [48] and the rest were denoted as hypothetical (data not shown).

In this way, all chromosomal genes were assigned to the categories mentioned, as shown in Figure 1 for the comparison of *S. meliloti *with *A. tumefaciens*. This approach gives a panoramic view about shared and unshared genes with other rhizobial species. Additional file 1 shows the categories for the other comparisons.

A schematic representation of rhizobial chromosomes was obtained relative to the classification of the genes. A high proportion of chromosomal predicted orthologs of the analyzed species were syntenic; however, in the *A. tumefaciens *linear, *M. loti *and *B. melitensis *I chromosomes, plasmidic homologs and species-specific genes were particularly abundant.

##### Gene organization in operons and microsyntenic regions in Rhizobiales

A relationship between predicted orthologs and operons was also explored. For *S. meliloti *(in comparison with *A. tumefaciens*), one half of the syntenic genes was found organized in 303 syntenic operons, a half of the total predicted operons (606); similar proportions were found for the *A. tumefaciens *circular and *B. melitensis *I chromosomes. In the *A. tumefaciens *linear, *B. melitensis *II and *M. loti *chromosomes, the proportion was about 21% (Table 1). The first two can be considered as accessory chromosomes and the last is the largest chromosome of the analyzed species. Non-syntenic genes were found organized in operons in a very small proportion in all analyzed Rhizobiales (Table 1).

A relevant level of operon conservation was found. In the comparison of *S. meliloti *with *A. tumefaciens*, 50% of the syntenic genes organized in operons were in identical operons, and taken together with those in similar operons (differing by one or two genes), 82% of total syntenic genes were in conserved operons. Similar proportions were found for the other comparisons (data not shown).

The operons formed with plasmidic homologs constituted a small fraction of the predicted operons (Table 1). Also, a reduced proportion was found for species-specific operons, except for *Ml*. Finally, mixed operons were present in a higher amount and ranged from 37 to 64% of the predicted operons (Table 1). This category revealed the highest rate of chromosomal rearrangements in these organisms. The mixed operons contained 17% of the syntenic genes, on average.

**Figure 2:**
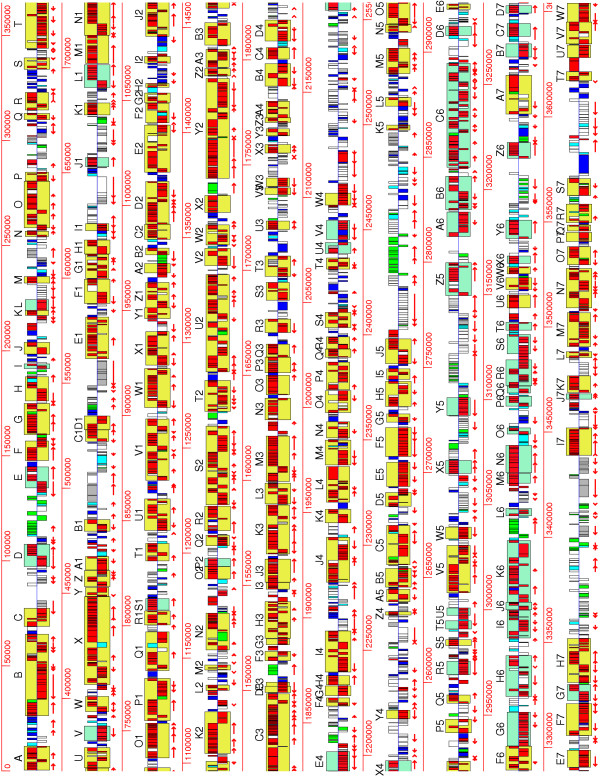
Synteny histogram of the *S. meliloti *chromosome in comparison to *A. tumefaciens *chromosomes. Red bars, syntenic genes. Framed with yellow boxes, microsyntenic regions with the *A. tumefaciens *(*At*) circular chromosome. Framed with light green boxes, microsyntenic regions with the *At *linear chromosome. Microsyntenic regions are denoted by letters (and numbers) in progressive order. Dark blue bars, non-syntenic genes with the *At *circular chromosome. Light blue bars, non-syntenic genes with the *At *linear chromosome. Green bars, homologs in plasmids. Gray bars, species-specific genes. White bars, homologs with other Rhizobiales chromosomes. Direction of transcription is denoted by upper (plus) or lower (minus) positions in respect to the central line. Predicted operons are denoted by red arrows. Scale in bp.

**Figure 3:**
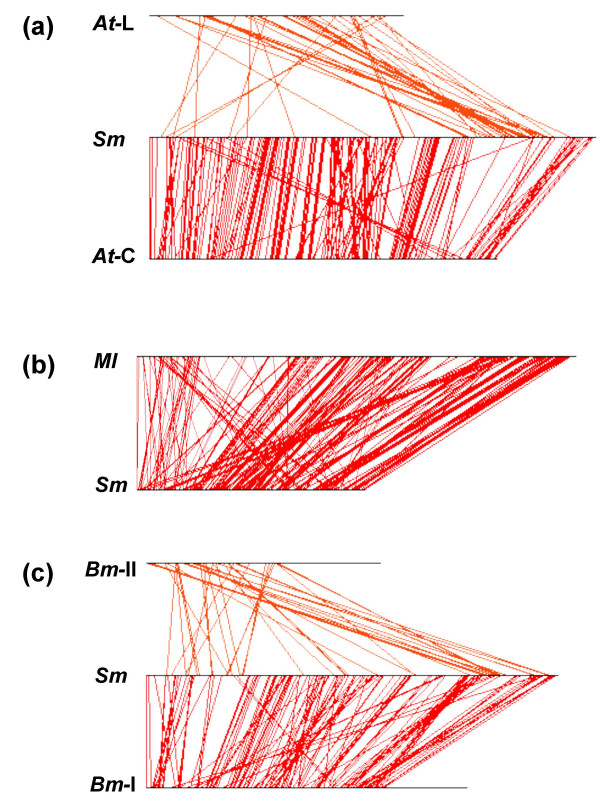
Schematic rearrangement of microsyntenic regions among *S. meliloti*, *A. tumefaciens, M. loti *and *B. melitensis *chromosomes. Panels: (a), *S. meliloti *chromosome (middle line) compared with *A. tumefaciens *circular (bottom line) and linear (upper line) chromosomes. (b), *S. meliloti *chromosome (lower line) compared with *M. loti *(upper line) chromosome. The *M. loti *chromosome was segmented in two fragments to maximize colinearity (see **Methods**). (c), *S. meliloti *chromosome (middle line) compared with *B. melitensis *chromosome I (bottom line), and II (upper line). *oriC *of *Bm *I was inverted to obtain maximal colinearity (see **Methods**). Red lines (orange for *At*-L and *Bm*-II chromosomes) represent the initial positions of the microsyntenic regions in each of the species analyzed.

Syntenic genes were found in clusters and were assigned to microsyntenic regions (see **Methods**). When comparing *S. meliloti *with both *A. tumefaciens *chromosomes, 205 regions were common. In particular, *At*-C regions in common with *Sm *chromosome were found along all the chromosome, except in the third quarter (Figure 2). The third quarter had colinearity with *At*-L (Figure 2, see also Figure 3 panel a). In the other comparisons, a similar amount of common regions were observed (Table 1, see also Additional files 2 and 3). 146 regions were shared in *Sm, At*, and *Ml *and 94 regions were common to the four Rhizobiales. About 90% of syntenic genes were located in the microsyntenic regions (Table 1).

When *At*-C syntenic genes were compared with *Sm *chromosome, a high level of colinearity was found (see Additional file 4 panel a). In the case of *Ml*, the synteny was disrupted possibly due to a conflicting annotation; for *Bm*-I, an inverse colinearity was obtained, possibly by *oriC *inversion relative to *Sm *(see Additional file 4 panels b and c, respectively). In regard to the rearrangement of microsyntenic regions, an extensive chromosomal colinearity was observed in long tracts. For example, in the comparison of *Sm *and *At *circular chromosomes, 93 microsyntenic regions were colinear, 24 almost colinear, 18 with drastic changes, and 13 inverted. The schematic representation is shown in Figure 3 panel a, lower part. In the comparison with the *At *linear and *Bm*-II chromosomes, highly rearranged structures were found (Figure 3 panel a, upper part and panel c, upper part). Rearrangement of microsyntenic regions on the chromosomes of *M. loti *(Figure 3 panel b) and *B. melitensis *I (Figure 3 panel c, lower part), showed a high level of colinearity when the conflicting annotation and the *oriC *inversion, respectively, were modified (see **Methods**).

Syntenic genes were organized mainly at two levels: operons and microsyntenic regions. Syntenic operons were as abundant as mixed operons. Extensive blocks of chromosomal colinearity were found, despite rearrangements.

##### Synteny and insertion sequences, horizontal gene transfer and codon usage

The mobile elements play an important role in chromosomal rearrangement. To determine how these elements were dispersed among microsyntenic regions, insertion sequence (IS) and transposase locations were analyzed. The *S. meliloti *chromosome contains 51 IS and 68 transposases belonging to diverse families [21]. Of the total transposases, 40 were found with homologs in plasmids, 20 were common with other rhizobial chromosomes, 6 were denoted as species-specific and 2 were common with the *A. tumefaciens *linear chromosome. Only 14 pairs of IS/transposases (27% of the total) were found inside microsyntenic regions.

**Figure 4:**
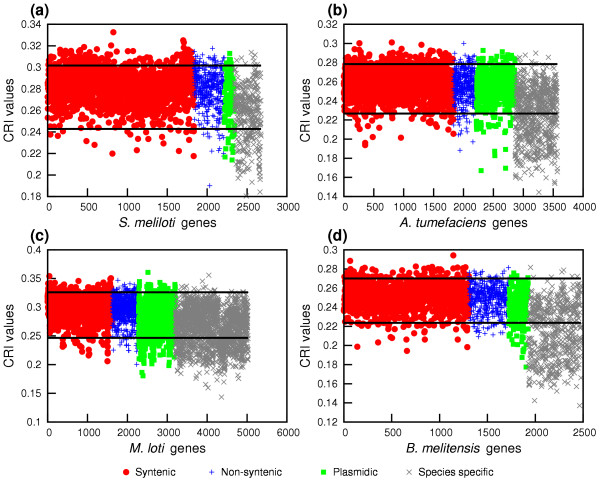
Codon richness index (CRI) for rhizobial genomes. All gene classifications were based on comparisons against *S. meliloti*, unless explicitly stated otherwise. Panels: (a), *S. meliloti *(compared with *A. tumefaciens*). (b), *A. tumefaciens*. (c), *M. loti*. (d), *B. melitensis*. CRIs were calculated according to the method described by Medrano-Soto et al. [27]. Symbols: red circles, syntenic genes. Blue plus signs, non-syntenic. Green squares, homologs in plasmids. Gray crosses, species-specific genes. Horizontal lines denote the species-specific thresholds for low and high CRI.

We assessed the influence of horizontal gene transfer (HGT) on the genomic structure of rhizobial genomes (see **Methods**). Table 2 shows calculated HGT events for each of the classes syntenic, non-syntenic and plasmid homologs. Even though syntenic genes were in the largest class analyzed, they displayed the lowest number of HGT events (Table 2). Predicted HGT rates of non-syntenic genes were 2 to 8 times higher than those of syntenic genes. On the other hand, unlike any other gene class, species-specific genes had the strongest bias toward a low codon richness index in the four Rhizobiales (denoted with crosses, Figure 4 panels a to d). No significant differences were found for syntenic or non-syntenic genes in each organism.

**Table 2:** Horizontal gene transfer prediction for *Rhizobiales*.

A. Gene class	HGT events*	*S. meliloti *genes	HGT ratio
Syntenic	5	1837	0.0027
Non-syntenic	8	378	0.0212
Plasmidic	3	129	0.0233
B. Gene class		*A. tumefaciens *genes	
Syntenic	1	1837	0.0005
Non-syntenic	1	378	0.0026
Plasmidic	0	649	0.0000

C. Gene class		*M. loti *genes	
Syntenic	7	1624	0.0043
Non-syntenic	6	655	0.0092
Plasmidic	11	924	0.0119

D. Gene class		*B. melitensis *genes	
Syntenic	6	1311	0.0046
Non-syntenic	3	414	0.0072
Plasmidic	5	204	0.0245

*Calculated by using the method described in Medrano-Soto *et al*. [27].

#### 2) Synteny in Enterobacteriales (gamma proteobacteria)

To assess the adequacy of applying our synteny analysis to another bacterial clade, we chose two members from the Enterobacteriales (gamma proteobacteria), the closely related *E. coli *and *S. typhimurium *genomes and defined their orthologous genes. These organisms contained 3092 predicted orthologs and 95% of them (2943) were syntenic. An extensive chromosomal colinearity with few rearrangements was found (data not shown).

Also, we chose a more phylogenetically distant species, *Erwinia carotovora *subsp. atroseptica to compare with *E. coli*. Their genomes comprise 4254 and 4477 genes, respectively. They shared 2477 orthologous genes and these represented about half the total genes in each chromosome. In the detection of synteny between these organisms, 1993 genes (80.4% of the orhologs) fulfilled our requirement and the rest, 484, were classified as non-syntenic. When the genes were assigned to microsyntenic regions, 230 regions were found and contained 92.8% (1849) of total syntenic genes and the rest, 7.2% (144 genes), were detected in the non-conserved tracts (see Additional file 5). 172 non-orthologous genes also formed part of microsyntenic regions. The 230 non-conserved regions contained 2233 genes.

To define the conservation of orthology and synteny of Enterobacteriales genomes in comparison with the Rhizobiales, we compared the *S. meliloti *and *E. coli *chromosomes. We found 777 predicted orthologs between them, a proportion that represents only a third of the genes shared in the Rhizobiales. By visual examination no synteny was apparent between the *Sm *and *E. coli *chromosomes (see Additional file 4 panel d). With an algorithm, only 198 syntenic genes were assigned to 65 microsyntenic regions.

#### 3) Sequence analysis of the chromosomal predicted orthologs

**Figure 5:**
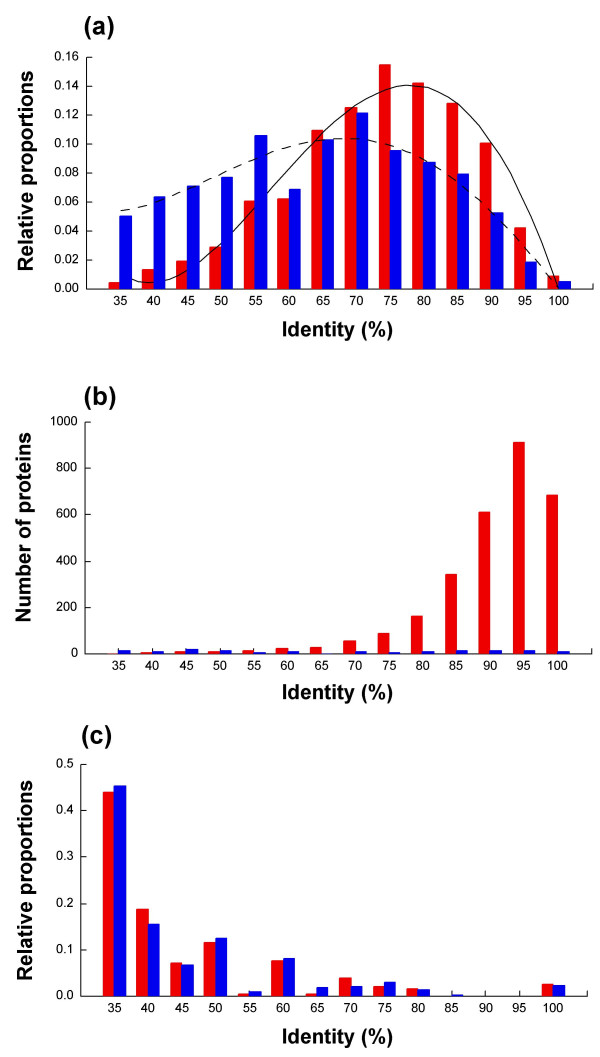
Sequence identity distribution of chromosomal predicted orthologs. Panels: (a), syntenic and non-syntenic products from the *S. meliloti-A. tumefaciens *(both chromosomes) comparison. Y-axis, relative proportions. (b), syntenic and non-syntenic products from the *E. coli-S. typhimurium *comparison. Y-axis, number of proteins in each range. (c), syntenic and non-syntenic products from the *S. meliloti-E. coli *comparison. Y-axis, relative proportions. Red bars, syntenic products. Blue bars, non-syntenic products.

To determine the level of sequence identity among chromosomal predicted orthologs of the Rhizobiales, distribution curves of their translation products were obtained. Two different types of curves were found. By comparing *S. meliloti *with both *A. tumefaciens *chromosomes, the syntenic products presented a Gaussian distribution, with a tendency to high identity levels (asymmetry coefficient g1 = -0.48, significant at P < 0.001) (Figure 5 panel a) and a mean value of 71.3%. The non-syntenic products showed a non- asymmetric distribution (g1 = -0.09) with a lower mean value (61.9%) (Figure 5 panel a). Similar curves were found for the chromosomal predicted orthologs of the other comparisons (Additional file 6 panels a and b, for *Sm-Ml *and *Sm-Bm *comparisons, respectively). When *At *replicons were separately compared with *Sm*, syntenic products of both *At*-C or *At*-L showed a similar bias toward high identity levels; in contrast, non-syntenic products of *At*-L showed a strongly deviated distribution to low identity values (data not shown). The tendency of syntenic products to higher identity levels reflects not only restriction to change but also functional constraints, possibly due to an essential character. Conversely, the lower identity levels of non-syntenic products represent lower restrictions to change and higher functional versatility.

A comparison of *E. coli *and *S. typhimurium *genomes (belonging to the Enterobacteriales) was performed. A very asymmetric distribution curve with a tendency to high identity levels for syntenic products was obtained (Figure 5 panel b); it remarkably resembled that obtained in the comparisons of syntenic products of the Rhizobiales. Interestingly, in the comparison of *S. meliloti *with *E. coli *both chromosomal non-syntenic and syntenic products had asymmetric curves with strong tendency to low identity levels (Figure 5, panel c).

**Figure 6:**
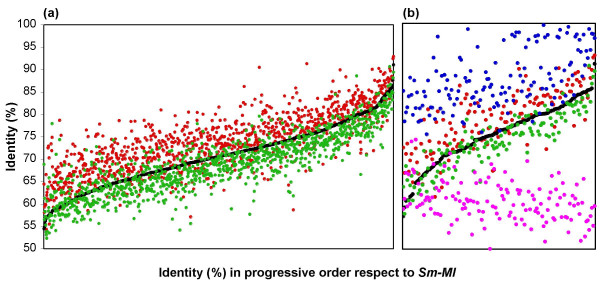
Sequence identity analysis of common syntenic genes. Panels: (a), in the four Rhizobiales. Comparisons: Red dots, *S. meliloti-A tumefaciens*. Green dots, *S. meliloti-B. melitensis*. (b), in Rhizobiales and Enterobacteriales. Comparisons: Red dots, *S. meliloti-A tumefaciens*. Green dots, *S. meliloti-B. melitensis*. Magenta dots, *E. coli-S. meliloti*. Blue dots, *E coli-S. typhimurium*. Reference line (in black) is the identity percentage of *S. meliloti-M. loti *syntenic genes in progressive order.

To obtain a complete view of sequence variation of chromosomal predicted orthologs in the four Rhizobiales, either syntenic or non-syntenic genes were graphed in relation to their identity levels in comparison with *S. meliloti*. Figure 6 panel a shows the sequence identity of 1038 common syntenic genes in the four Rhizobiales, for *Sm-At*, *Sm-Ml *and *Sm-Bm *comparisons. We referred the comparison to the identity of *Sm-Ml *genes ordered progressively. We found that each comparison showed a particular clustering, probably related to the phylogenetic distances between these organisms. The Pearson correlation coefficients were r = 0.81 and r = 0.88 for *Sm-Bm *and *Sm-At *comparisons, respectively. In the clustering of 98 non-syntenic genes common to the four Rhizobiales (data not shown), lower correlation coefficients were obtained (r = 0.71 and r = 0.79, for *Sm-Bm *and *Sm-At *comparisons, respectively). In Figure 6 panel b the sequence identity of 140 common syntenic genes in the Rhizobiales and Enterobacteriales are shown. While the comparison for Rhizobiales follows a similar tendency to the previously observed, for *E. coli *and *Salmonella *there is a higher relatedness level. However, when comparing *S. meliloti *and *E. coli *a different tendency with very low identity level is observed. A high level of clustering among closely related species belonging to the same subdivision is visible and also, a tendency to reduced relatedness in members of different subdivisions; that is, synteny is a common trait for members of each subdivision.

To determine the meaning of sequence differences we analyzed the translated syntenic product ArgC, which participates in the arginine biosynthetic pathway. The alignment presented in Additional file 7 panel a shows 111 positions with identical residues and a range from 33 to 113 different residues, particular for each of the species compared. However, sequences from *Brucella melitensis *and *Brucella suis *showed only one difference between them (not shown). Synteny is almost absolute in these organisms (Additional file 4 panel e). Changing residues (possibly species signatures) were dispersed along the sequences and varied according to the identity level.

To determine more comprehensively sequence differences and similarities in Rhizobiales (alpha proteobacteria) and Enterobacteriales (gamma proteobacteria), we selected five organisms from each: *S. meliloti, A. tumefaciens, M. loti, B. melitensis *and *R. palustris *for the first and *E. coli, S. typhimurium, S. flexneri, Buchnera *sp. and *E. carotovora *for the last. We chose some syntenic products from the arginine biosynthetic pathway, namely ArgB, ArgC, ArgD, ArgF, ArgG, and ArgH. The alignment belonging to ArgC is shown in Additional file 7 panel b. As can be observed, there are sequence identities and differences among species from the same order ("species signatures"), but also similarities between species of different orders, albeit at smaller level. Additionally, we found an interesting pattern: proteins from Enterobacteriales showed an almost uniform level of sequence identity and similarity (about 50 and 70%, on average, respectively), however, sequences from Rhizobiales showed a clear increasing tendency, from 25 to 62% in identity, and from 44 to 81% in similarity (see Additional file 7 panel c). These different profiles possibly are related with the particular conditions of the niches occupied by these organisms.

#### 4) Physical characteristics of the translated syntenic genes

**Figure 7:**
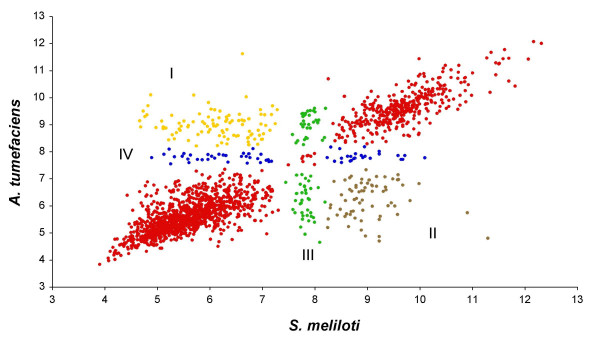
Theoretical isoelectric points (pI) of the *S. meliloti-A. tumefaciens *syntenic products. Dots represent translated products. Red dots, products on the diagonal. Yellow, dots, sector I. Brown dots, sector II. Green dots, sector III. Blue dots, sector IV (see **Results**). Scales in pH units.

Molecular weight (MW) and isoelectric point (pI) are the main traits for proteomic comparisons. To determine whether syntenic and non-syntenic genes could present differential protein characteristics, we graphed pairs of translated predicted orthologs for both MW and pI parameters. In all comparisons, MW graphs showed lower dispersion than pI ones. Figure 7 shows the pI graph for the *S. meliloti-A. tumefaciens *comparison (*Sm-M. loti *and *Sm-B. melitensis *comparisons are in Additional file 8 panels a and b, respectively). A large group of proteins (82% in each comparison) was located on the diagonal. The correlation coefficient for this group was r = 0.96 in all comparisons. However, the rest of the predicted proteins showed differential pI's and were assigned to sectors (Figure 7). Sector I had acidic proteins in *S. meliloti *and basic proteins in the organism compared with it. Sector II had basic proteins in *S. meliloti *and acidic in organism compared to it. In sector III were neutral proteins in *S. meliloti *and covered all pI range in each of the organisms compared. Proteins in sector IV were neutral in the comparing organism and covered all pI ranges in *S. meliloti*. In Additional file 9 there is a summary of pI variability of common syntenic products from comparisons with the chromosomes of *S. meliloti, A. tumefaciens *and *M. loti*. About 75% of products showed similar pI and 14% and 8% presented high and low variation, respectively. High level is defined as pI variation from acid in one or two organisms and basic in the other, and viceversa. Low level is defined as pI variation from neutral to acid or basic (Additional file 9). Proteins of the sectors mainly corresponded to the functional categories of energy generation, post-translational modification, and transport. In the case of non-syntenic products, a pattern similar to that described above was found (see Additional file 10). Proteins in the diagonal were the most conserved group with subtle pI changes possibly responding to species adaptation, whereas those with deviated pI's may represent a group with higher functional versatility.

Since the functional categories mentioned above for sectors are known to often interact with the cell membrane, a membrane prediction for all syntenic products from the *S. meliloti-A. tumefaciens *circular chromosomes comparison was assessed. Strikingly, 790 syntenic products were predicted to contain membranal segments. This amount represents almost all membranal proteins coded in the *S. meliloti *chromosome, considering that bacterial genomes have 18–28% membranal proteins [49-51]. About 70% of predicted membranal proteins with assigned function belonged to transport, energy generation, post-translational modification, cofactor synthesis, amino acid metabolism and central intermediary metabolism categories (Additional file 11). There are reports about membrane-interacting proteins with functions such as amino acid and cofactor biosynthesis and central intermediary metabolism [52-56].

To determine whether the charged amino acid residues were clustered in proteins from the sectors described above, we selected several proteins from each. The residues determining radical changes in pI were observed scattered along the sequences (data not shown).

#### 5) Functional roles and linkage of the chromosomal predicted orthologs

**Figure 8:**
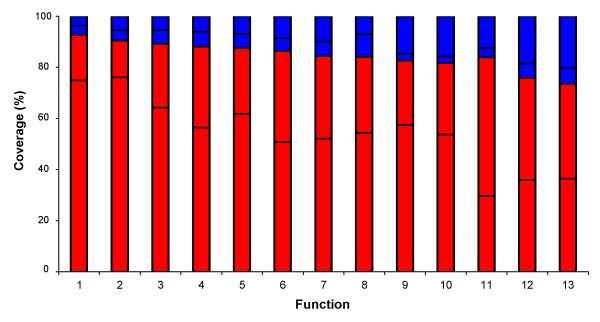
Coverage of fuctional classes with syntenic and non-syntenic genes in the *S. meliloti-A. tumefaciens *comparison. X-axis, functional classes: 1) Transcription, 2) Translation, 3) Fatty acid and phospholipid metabolism, 4) Cell envelope, 5) Biosynthesis of cofactors, prosthetic groups and carriers, 6) Purine, pyrimidine, nucleoside and nucleotide metabolism, 7) DNA metabolism, 8) Amino acid metabolism, 9) Cellular processes, 10) Energy metabolism, 11) Transport and ATP binding proteins, 12) Regulatory functions, 13) Central intermediary metabolism. Red bars, lower fraction: syntenic genes in the four Rhizobiales; upper fraction, syntenic genes in the *S. meliloti-A. tumefaciens *comparison. Blue bars, lower fraction: non-syntenic genes in the four Rhizobiales; upper fraction: non-syntenic genes in the *Sm-At *comparison. Y-axis, % of coverage.

To define the metabolic participation of the chromosomal predicted orthologs, a functional classification was made with chromosomal syntenic and non-syntenic genes. As shown in Figure 8, syntenic genes of *S. meliloti-A. tumefaciens *chromosomes contained a high proportion of the most important house-keeping functions. The relative proportion of non-syntenic genes grew with decreasing functional essentiality, for example transport and binding proteins, cellular processes and regulatory functions (Figure 8). Furthermore, the syntenic products covered 85% of the main metabolic pathways as defined in MetaCyc for *S. meliloti *(see Additional file 12). Similar results in functional coverage were obtained with the other comparisons (see Additional file 13 panels a and b). Common syntenic genes in the four Rhizobiales also included a large fraction of the house-keeping functions (lower segment of the red bars, Figure 8). In the case of the comparison between *E. coli *and *E. carotovora*, syntenic genes also covered a high proportion of essential functions, however, the first two positions were occupied by cofactor and nucleotide synthesis (see Additional file 13 panel c). In this regard, it is important to note that *E. coli *and *E. carotovora *possess no large plasmids.

**Figure 9:**
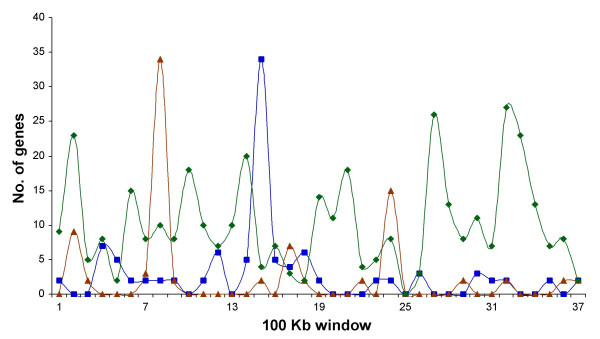
Distribution of syntenic genes (in regions) by functional superclasses in the *S. meliloti *chromosome. Blue squares, informational processes. Red triangles, cellular processes. Green diamonds, operational functions. For distribution only microsyntenic regions (from the *S. meliloti-A. tumefaciens *comparison) with at least two genes belonging to a given class were considered.

When functional classes of genes in the microsyntenic regions were divided into Informational, Operational and Cellular processes superclasses and graphed for the *S. meliloti *chromosome with a 100 Kb window, an interesting pattern was observed (Figure 9), with the majority of the peaks belonging to a superclass matching with valleys of the other(s). This could represent functionally specialized blocks of chromosomal tracts, which were part of the ancestral rhizobial chromosome. For instance, the existence of genomic domains is accepted in eukaryotes [57].

The ProLinks program was used to determine how the chromosomal predicted orthologs are functionally related. Functional links were calculated for all genes in the *S. meliloti *and *E. coli *chromosomes and then correlated to their neighbors. For the *S. meliloti-A.tumefaciens *comparison, the microsyntenic regions presented, on average, 3.68 connections per node (a protein in a network), almost twice the value obtained in the non-conserved regions (2.06). From 205 microsyntenic regions, 104 had functional networks; in the case of non-conserved regions, only 35 presented networks. Networks with less than 6 connections were omitted. The networks of microsyntenic regions presented 1057 syntenic genes and from these 795 (75.2%) were organized in operons. 99 non-syntenic genes were in the networks of the non-conserved tracts, and only 56 (56.5%) were in operons. In the case of the synteny comparison between *E. carotovora-E. coli *(Enterobacteriales), 230 microsyntenic regions were obtained and from these, 161 presented functional networks, with a connectivity average of 5.33 connections/node. The non conserved regions with networks were 71 with a connectivity average of 2.04 connections/node. From 1497 syntenic genes in the networks, 1106 (73.8%) formed part of operons. Network connectivity obtained in *S. meliloti *and *E. coli *is shown in the graph of Additional file 14 (panels a and b, respectively). There is a striking difference in connectivity level in the networks from syntenic (gray bars) or non-conserved regions (black bars). The connectivity levels in syntenic vs non-conserved regions in both organisms were similar using the STRING program (data not shown).

## Discussion

The comparative genomic analysis reported here was useful in finding interesting gene properties. Orthologs with conserved replicon and neighborhood were the principal component of the chromosomes. Compared with non-syntenic genes, syntenic ones had higher identity levels, lower horizontal gene transfer (HGT) rates, showed strongly organized structures as operons and microsyntenic regions and a relative absence of mobile elements. Thus, the syntenic genes can be considered as the chromosomal backbone of the order. Plasmidic homologs were scattered on the chromosomes, and higher HGT rate and linkage to transposases support their extrachromosomal origin. Species-specific genes had the lowest codon richness index, and possibly were acquired in the evolutionary history of each of the species.

In this way, a rhizobial chromosomal origin can be envisioned. The chromosomal orthologs were the gene set derived from the common cenancestor. From these, syntenic genes conserved a relative chromosomal order (and operonic organization) and encode the essential functions of the cell; non-syntenic genes lost the clustering and possibly some came from HGT events. The plasmidic homologs were obtained possibly by mobilization throughout replicons, a nonrare process in the rhizobial phylogenetic branch. The species-specific genes represent the particular gene set of the species and are the most intriguing group due to their unknown functional roles and origin. Work with members of the last group will help to define traits not shared with other species.

The Rhizobiales species analyzed showed a striking proportion of orthologous genes, mainly chromosomal syntenic; non-syntenic genes were found in lower proportion. A large fraction of the first class was common in the four organisms. Syntenic genes had a strong tendency to form operons and almost all were clustered in microsyntenic regions. Additionally, these operons were conserved in pairs of organisms. Therefore, a strong restriction for chromosomal rearrangement is visible. Given that these organisms cover a wide spectrum of environmental distributions, from plant rhizosphere to animal host, the conserved chromosomal tracts may be important to determine the metabolic properties common to the order. Similar results were observed in the Enterobacteriales comparison. In a recent report, using computational inference, Boussau *et al*. [58] proposed a common ancestral set of about 3000 genes for proteobacterial genomes.

Although rhizobial genomes shared common traits, important differences were also observed. For example, abundance of plasmidic homologs and species-specific genes in *A. tumefaciens *(linear), *B. melitensis *II and *M. loti *chromosomes confirmed their complex evolutionary histories [20,22,59,60]. The *M. loti *chromosome presents intensive incorporation of foreign genes by horizontal transfer, such as those belonging to the symbiotic island [59]. In regard to species-specific genes, a large number presented low codon richness index. This category will be reduced with incorporation of other rhizobial genomes into the databases. For example, *R. leguminosarum *biovar *viciae *3841, *R. tropici *PRF81, *R. sp*. ANU265 and *R. etli *CFN42 genomes soon will be available [1]. Mobilization elements participate in chromosomal rearrangement and are abundant in Rhizobiales. These elements can decompose the microsyntenic regions where they are. In this way, all synteny approaches consist of snapshots in chromosomal evolution.

From the comparison of sequence identities among chromosomal predicted orthologs in the rhizobial species analyzed, an interesting characteristic was the differential distribution curves obtained. The asymmetric curves of syntenic products, deviated to high identity levels (with peaks at 70–75%, Figure 5 panel a and Additional file 6 panels a and b), possibly reveal their essentiality and can be compared with those from gamma proteobacteria with high identity (with peak at 95%, Figure 5 panel b). Conversely, non-syntenic genes had curves with lower sequence identity levels reflecting a higher functional versatility. In the case of *S. meliloti*-*E. coli *comparison, the identical curves for syntenic and non-syntenic genes with the majority at very low identity values (35%, Figure 5 panel c) reflect their greater phylogenetic distances.

The high identity of syntenic genes indirectly reveals their essential character; for the non-syntenic genes the low identity could represent adaptability to the ecological niche of the species. The identity relatedness in the syntenic genes among rhizobial species (Figure 6 panel a) revealed a cohesively evolved group; additionally the sequence differences were reflected in the theoretical pI plots of the proteins encoded by these genes, with a majority in the diagonal and the rest in sectors with strong pI deviation. Species signatures of the sequences (see Additional file 7) showed a differential level of changed residues and these could represent functional adaptation to a niche; this proposal is supported with the almost identical sequences of *B. melitensis *and *B. suis*. On the other hand, invariant peptides perhaps contribute to structural conformation [61]. Experiments in progress in our lab will determine the validity of our proposal. Recently, a conservative change which altered the function of the transcriptional regulator BosR [62], and pathogenic differences in enteric bacteria due to the expression of PmrD regulators with divergent sequences [63] were reported.

A typical trait of Enterobacteriales is its pathogenic character, and this means a very intimate, frequent contact with their hosts and almost constant, homogeneous conditions: the extreme case is that of *Buchnera *sp., an aphid-obligate symbiont. In contrast, Rhizobiales are commonly found in the soil in saprophytic living style, and occasionally they associate with hosts, and therefore face more heterogeneous, variable environments. These features were reflected in the sequence comparisons of proteins from the arginine biosynthetic pathway (see Additional file 7). The syntenic gene organization was different in the Rhizobiales compared with Enterobacteriales, however it is important to note the high orthology and synteny degrees between members of each clade, the essentiality of functions covered by the syntenic genes and the high functional linkage in the microsyntenic regions in each chromosome (see below). From the previous observations we can obtain a general trend: bacterial clades present a particular chromosomal gene arrangement and such plasticity possibly was selected in relation to the niches occupied by these organisms.

As have others, some time ago we found a proteomic bimodal distribution when MW and pI were plotted for rhizobial gene products. The main fraction was located at acid and basic pI's, in a shape resembling butterfly's wings and the body, at neutral pI, presenting a low number of proteins. Recently Knight *et al*. [64], in a vast genomic study, reported similar results and additionally, related proteome similarities to shared metabolic features. In this respect, we plotted pI of syntenic products from pair comparisons of studied species in order to detect proteomic similarities in these organisms. It was possible to analyze the pI variability in each of the common syntenic products; however, it is necessary to carry out experimentation to determine the biological role of pI variation. The overrepresented membranal fraction in the syntenic products must be further explored to determine whether they respond to different extracellular/intracellular signals. A rapid evolution for the membranal proteomic fraction was suggested in the same study [64].

The high proportion of syntenic operons in the microsyntenic regions and the duplicated connections per gene in the network-forming regions (in regard to non-conserved regions), in Rhizobiales but also in Enterobacteriales, supported the functional linkage and interaction of these genes in the conserved tracts (see Additional file 14), and this is a factor which could help to define the role of selective pressure in maintaining the gene order.

Functional characterization of the predicted orthologs deepened our understanding of their cellular roles. In both Rhizobiales and Enterobacteriales, a clear essentiality was observed for chromosomal syntenic genes, agreeing with their sequence restrictions for change. Non-syntenic genes, on the other hand, appeared abundant in functions granting metabolic versatility to the cell. By calculating nonsynonymous/synonymous substitution rates, other authors have shown that in bacteria most conserved genes cover the essential functions of the cell [65].

## Conclusion

Our synteny analysis defined a multi-level gene organization in the bacterial chromosome. Restriction of sequence variation in these genes, with clear essential functional roles, appeared extended to the conservation of chromosomal arrangement. In this way, synteny possibly has an important biological significance in these organisms.

## Methods

### Identification of orthologs

Available genome sequences of the fast growing Rhizobiales, *S. meliloti *1021 [accession number GenBank: NC_003047], *A. tumefaciens *C58 (Cereon) [GenBank: NC_003062 and NC_003063], *M. loti *MAFF303099 [GenBank: NC_002678] and *B. melitensis *16 M [GenBank: NC_003317 and NC_003318] were obtained from the Genome division of the NCBI Entrez system [66]. Genomes of *E. coli *K12, *S. typhimurium *LT2 and *Erwinia carotovora *subsp. atroseptica SCRI1043 [accession numbers GenBank: NC_00913NC_003197, and NC_004547], were used for synteny and functional analysis. The genome of *B. suis *1330 [accession numbers GenBank: NC_004310 and NC_004311 for chromosomes I and II, respectively] was used for synteny and sequence identity analysis. Genomes of *Bradyrhizobium japonicum *USDA110 [accession number GenBank: NC_004463] and *Rhodopseudomonas palustris *CGA009, also belonging to the alpha proteobacteria, were not considered in the main analysis because of their more distant phylogenetic relationship with the fast-growing rhizobia. To obtain a comprehensive view of shared genes among Rhizobiales, we differentiated genes by orthology and their presence in the same or different replicons in the analyzed species. Chromosomal orthologs were assigned by the best bidirectional hit between pairs of organisms, using the Fasta34 program [67]. Unidirectional best hits (homologs) were considered to cover complete chromosomal gene number (see **Results**) and for detection of chromosomal genes with plasmidic homologs. Parameters were: an identity of at least 50%, overlapping by at least in 150 nt and an expectance (E) score of <10^-3^. The base organism for comparisons was *S. meliloti *1021. GenBank accession numbers of proteins used for alignments in the order ArgB, ArgC, ArgD, ArgF, ArgG and ArgH, were as follows. Rhizobiales. *R. palustris*: NP_945982.1, NP_947833.1, NP_950107.1, NP_950106.1, NP_945745.1, NP_950077.1; *B. melitensis*: NP_541250.1, NP_540088.1, NP_540538.1, NP_540537.1, NP_540787.1, NP_539004.1; *M. loti: *NP_105609.1, NP_108547.1, NP_106269.1, NP_106270.1, NP_105253.1, NP_104594.1; *A. tumefaciens: *NP_353412.1, NP_354256.1, NP_353456.1, NP_353457.1, NP_355604.1, NP_357013.1; *S. meliloti: *NP_384545.1, NP_385346.1, NP_384623.1, NP_384624.1, NP_387315.1, NP_386753.1. Enterobacteriales. *Buchnera sp*.: NP_239886.1, NP_239885.1, NP_240341.1, NP_240186.1, NP_239887.1, NP_239888.1; *E. coli: *NP_418394.1, NP_418393.1, NP_417818.1, NP_414807.1, NP_417640.1, NP_418395.1; *E. carotovora*: YP_048320.1, YP_048319.1, YP_052152.1, YP_048510.1, YP_048232.1, YP_048321.1*S. typhimurium: *NP_457937.1, NP_457938.1, NP_458434.1, NP_458882.1, NP_457671.1, NP_457936.1; *S. flexneri*: NP_838925.1, NP_838926.1, NP_839526.1, NP_839629.1, NP_838682.1, NP_838924.1. All data sets are available on request.

### Procedures for detection of syntenic genes, microsyntenic region formation and operon similarity

To consider chromosomal orthologs as syntenic among pairs of organisms, at least two genes must remain contiguous in both chromosomes. Microsyntenic region formation and extension fulfilled the following criterion: a pair of predicted orthologs separated from at least one other by no more than three genes (from the rest of categories). The minimal region was formed by a stretch containing three syntenic genes. Operon prediction was performed as reported by Moreno-Hagelsieb and Collado-Vides [68]. Rearrangements were graphed using initial positions of the microsyntenic regions in each chromosome. The syntenic regions of *M. loti *and *B. melitensis *I chromosomes were graphed so as to increase the colinearity in these replicons. The *M. loti *chromosome was segmented into two halves at 3.5 Mb position and the fragment covering from 3.5 to 7.0 Mb was located in the first position and then both halves were aligned with microsyntenic regions of *S. meliloti*. In the case of *Brucella *chromosome I, the origin was inverted. Graphs were obtained using the GenVision program (DNAStar Inc., Madison, WI). For operon similarity calculation, a limit of three different genes in each operon was allowed. All data sets are available on request.

### Detection of horizontal gene transfer

Prediction of horizontally transferred genes in the Rhizobiales genomes was performed using the method described by Medrano-Soto *et al*. [27]. Briefly, it on based in similar gene length, maximum global protein identity, conflicting phylogenies and codon usage of xenologous genes. In the case of *A. tumefaciens*, this rate was calculated using sequences and annotation obtained from the U. of Washington Sequencing Project [45]. Species-specific (or orphan) genes were not considered by two reasons: (1) the lack of orthologs in other genomes precluded phylogenetic analysis, and (2) impossibility of correlating these genes to a synteny.

### Sequence comparison of chromosomal orthologs in Rhizobiales

The identity of peptidic sequences of chromosomal predicted orthologs were used to graph the distribution curves. The asymmetry of distribution curves, or skewness, was calculated by the asymmetry coefficient of Pearson (g1) as described elsewhere [69]. To correlate nucleotide sequence identities of the chromosomal predicted orthologs in the four rhizobial genomes, the gene identity of the predicted orthologs from the *S. meliloti-M. loti *comparison was graphed in progressive order. Then, corresponding predicted orthologs of the other comparisons were located at their corresponding identity percentages. Correlation coefficient values were calculated by the Pearson method. Plasmidic homologs and species-specific genes were not graphed because they have no counterparts in the pairs of analyzed genomes.

### Theoretical proteome and transmembranal protein prediction

Theoretical proteomes were obtained by calculating molecular weight and isoelectric point for each translated chromosomal predicted ortholog. Both parameters were estimated with the pI/MW prediction tool of the Laquip Proteomic Team page [70]. To determine the set of orthologs coding proteins predicted to interact with the cell membrane, we used the TMAP program, version 46 [71], available in the EMBOSS package (European Molecular Biology Open Software Suite, [72]). Correlation coefficient values were obtained with the Pearson method. Alignments were performed with ClustalW [73].

### Functional classification of chromosomal predicted orthologs

Chromosomal predicted orthologs were assigned to the functional classes used for *Agrobacterium tumefaciens *C58 in the U. of Washington genome report [45]. For the distribution of functions coded in the microsyntenic regions along the *S. meliloti *chromosome, classes were assigned into Operational (including amino acid, fatty acid, carbohydrate and nucleotide metabolism, energy generation, central intermediary metabolism, transport and cofactor synthesis), Informational (DNA metabolism, transcription, translation and regulatory functions), and Cellular processes (cell envelope, cell division, secretion and chemotaxis) superclasses. This grouping, except for Cellular processes, is similar to that of Rivera *et al*. [74]. For functional relationship inference, the ProLinks [34] and STRING databases [35] were used with permission. The confidence levels used were 0.6 and 0.9, respectively. Resulting networks with ProLinks with less than 6 links were omitted from the count. Networks were constructed with the Pajek Program (written by A. Vlado), version 1.02, available in the web [75]. Assignment of metabolic pathways was performed using the MetaCyc database [76], with permission.

## Authors' contributions

GG and AA performed the computer predictions. HP made the functional analysis, interpreted the data and wrote the paper. RD and MAV participated in the work design. AM-S made the HGT and CRI analysis. JM conceived and directed the project. All authors read and approved the final manuscript.

## Supplementary Material

Additional File 1 Schematic representation of the Rhizobiales chromosomes in comparison with *S. meliloti*, according to the gene classification of predicted orthologs and homologs. Panels: (a), *S. meliloti-A. tumefaciens *comparison. (b), *S. meliloti-M. loti *comparison. (c), *S. meliloti-B. melitensis *comparison. Red striped bars, syntenic genes with the organism in comparison. Blue striped bars, non-syntenic genes with the organism in comparison. White bars, homologs with other Rhizobiales chromosomes (for *S. meliloti*, compare with Fig. 1, white fraction). Green bars, homologs in plasmids. Gray bars, species-specific genes. In panels a and c, the *S. meliloti *chromosome shows syntenic and non-syntenic genes with both replicons of the organisms under comparison. Red striped bars, syntenic genes, lower fraction: with (a) *At*-C and (c) *Bm*-I chromosomes; upper fraction: with (a) *At*-L and (c) *Bm*-II chromosomes. Blue striped bars, non-syntenic genes, lower fraction: with (a) *At*-C and (c) *Bm*-I chromosomes; upper fraction: with (a) *At*-L and (c) *Bm*-II chromosomes. Click here for file

Additional File 2 Synteny histogram of *S. meliloti *in comparison with *M. loti *chromosome. Red bars, syntenic genes. Surrounded with yellow boxes, microsyntenic regions. Microsyntenic regions are denoted by letters (and numbers) in progressive order. Blue bars, non-syntenic genes. Green bars, homologs in plasmids. Gray bars, species-specific genes. White bars, homologs with other Rhizobiales chromosomes. Direction of transcription is denoted by upper (plus) or lower (minus) positions in respect to central line. Predicted operons are denoted by red arrows. Scale in bp. Click here for file

Additional File 3 Synteny histogram of *S. meliloti *in comparison with *B. melitensis *chromosomes. Red bars, syntenic genes. Surrounded with yellow boxes, microsyntenic regions with *B. melitensis *chromosome I (*Bm*-I). Surrounded with light green boxes, microsyntenic regions with *B. melitensis *chromosome II (*Bm*-II). Microsyntenic regions are denoted by letters (and numbers) in progressive order. Dark blue bars, non-syntenic genes with *Bm*-I. Light blue bars, nonsyntenic genes with *Bm*-II. Green bars, homologs in plasmids. Gray bars, species-specific genes. White bars, homologs with other Rhizobiales chromosomes. Direction of transcription is denoted by upper (plus) or lower (minus) positions in respect to central line. Predicted operons are denoted by red arrows. Scale in bp. Click here for file

Additional File 4 Synteny of Rhizobiales and Enterobacteriales. Panels: (a), *S. meliloti-A. tumefaciens *circular chromosomes comparison. (b), *S. meliloti-M. loti *comparison. (c), *S. meliloti-B. melitensis *chromosome I comparison. (d), *S. meliloti-E. coli *comparison. (e), *B. suis-B. melitensis *chromosomes I comparison. Red dots, syntenic genes. Blue dots, non-syntenic genes. Scales in bp. Click here for file

Additional File 5 Synteny histogram of *E. coli *in comparison with *E. carotovora *chromosome. Red bars, syntenic genes. Surrounded with yellow boxes, microsyntenic regions with *E. carotovora *chromosome Microsyntenic regions are denoted by letters (and numbers) in progressive order. Blue bars, non-syntenic genes. Gray bars, non-orthologous genes. Scale in bp. Click here for file

Additional File 6 Sequence identity distribution of chromosomal translated orthologs. Panels: (a), syntenic and non-syntenic products from the *S. meliloti-M. loti *comparison. (b), syntenic and non-syntenic products from the *S. meliloti-B. melitensis *(chromosomes I and II) comparison. Y-axis, relative proportions. Red bars, syntenic genes. Blue bars, non-syntenic genes. Y-axis, relative proportions. Click here for file

Additional File 7 Sequence alignments and data from the alignments of proteins from the arginine biosynthetic pathway in Rhizobiales and Enterobacteriales. Panels: (a), ArgC in Rhizobiales. Identical residues for aech position are marked with yellow. Least abundant residues for a given position are denoted with an specific color for each of the species: dark blue, differences in *R. palustris*; green, differences in *B. melitensis; *red, differences in *M. loti; *gray, differences in *A. tumefaciens*; violet, *S. meliloti*. (b), ArgC in Rhizobiales and Enterobacteriales. Identical residues for aech position are marked with yellow. Least abundant residues for a given position are denoted with an specific color for each of the species: Rhizobiales, same code of panel (a). Enterobacteriales: brown, *Buchnera*; pink, *E. carotovora*; blue, *S. typhimurium*. *E. coli *and *S. flexneri*, none. (c), data of the identity (*) and similarity (:*) in residues and in percentage (bold) of the alignments of the proteins in Rhizobiales and Enterobacteriales. Click here for file

Additional File 8 Theoretical isoelectric points (pI) of syntenic products. Panels: (a), *S. meliloti-M. loti *comparison. (b), *S. meliloti-B. melitensis *(chromosomes I and II) comparison. Dots represent translated products. Scales in pH units. Click here for file

Additional File 9 Summary of proteins with differential pI's from comparisons with chromosomes of *S. meliloti, A. tumefaciens *(circular) and *M. loti*. Click here for file

Additional File 10 Theoretical isoelectric points (pI) of nonsyntenic products. Panels: (a), *S. meliloti-A. tumefaciens *(both chromosomes) comparison. (b), *S. meliloti-M. loti *comparison. (c), *S. meliloti-B. melitensis *(both chromosomes) comparison. Dots represent translated products. Scales in pH units. Click here for file

Additional File 11 Functional categories of syntenic products of the membranal prediction of *S. meliloti- A. tumefaciens *(circular chromosome) comparison. Click here for file

Additional File 12 Metabolic pathways covered by syntenic products of the *S. meliloti-A. tumefaciens *comparison. Scheme belongs to the MetaCyc pathways for *S. meliloti *chromosome, used with permission. Highlighted with green, reactions covered with syntenic products. Click here for file

Additional File 13 Coverage of functional classes with syntenic and non-syntenic genes. Panels: (a), *S. meliloti*-*M. loti *comparison. Classes: 1) Translation, 2) Transcription, 3) Purine, pyrimidine, nucleoside and nucleotide metabolism, 4) Cellular processes, 5) Energy metabolism, 6) Cell envelope, 7) Fatty acid and phospholipid metabolism, 8) Biosynthesis of cofactors, prosthetic groups and carriers, 9) Transport and ATP binding proteins, 10) Amino acid metabolism, 11) DNA metabolism, 12) Regulatory functions, 13) Central intermediary metabolism. (b), *S. meliloti*-*B. melitensis *comparison. Classes: 1) Transcription, 2) Translation, 3) Cellular processes, 4) Biosynthesis of cofactors, prosthetic groups and carriers, 5) Cell envelope, 6) Energy metabolism, 7) Fatty acid and phospholipid metabolism, 8) Purine, pyrimidine, nucleoside and nucleotide metabolism, 9) Amino acid metabolism, 10) Transport and ATP binding proteins, 11) DNA metabolism, 12) Regulatory functions, 13) Central intermediary metabolism. (c), *E. coli-E. carotovora *comparison. Classes: 1) Biosynthesis of cofactors, prosthetic groups and carriers, 2) Purine, pyrimidine, nucleoside and nucleotide metabolism, 3) Translation, 4) Fatty acid and phospholipid metabolism, 5) Transcription, 6) Cellular processes, 7) DNA metabolism, 8) Energy metabolism, 9) Amino acid metabolism. 10) Cell envelope, 11) Regulatory functions, 12) Transport and ATP binding proteins, 13) Central intermediary metabolism. Note that order of classes is different to that in Fig. 8. Red bars, syntenic genes. Blue bars, non-syntenic genes. Click here for file

Additional File 14 Connectivity values from networks formed by microsyntenic and non-conserved regions in (a) *S. meliloti *(in comparison with *A. tumefaciens*) and (b) *E. coli *(in comparison with *E. carotovora*). Y-axis, connections per network. First syntenic networks, with 1060 (*S. meliloti*) and 810 (*E. coli*) connections, were omitted for clarity. Arranged in decrecent connectivity order. Gray bars, microsyntenic regions. Black bars, non-conserved regions. Successive networks, with connectivity values lower than 6, were omitted. Click here for file
