# Supplementary figures and images for: Evolutionary, structural and functional relationships revealed by comparative analysis of syntenic genes in Rhizobiales
Source: BMC Evol Biol. 2005 Oct 17;5:55. doi: 10.1186/1471-2148-5-55 (PMC1276791; doi:10.1186/1471-2148-5-55)

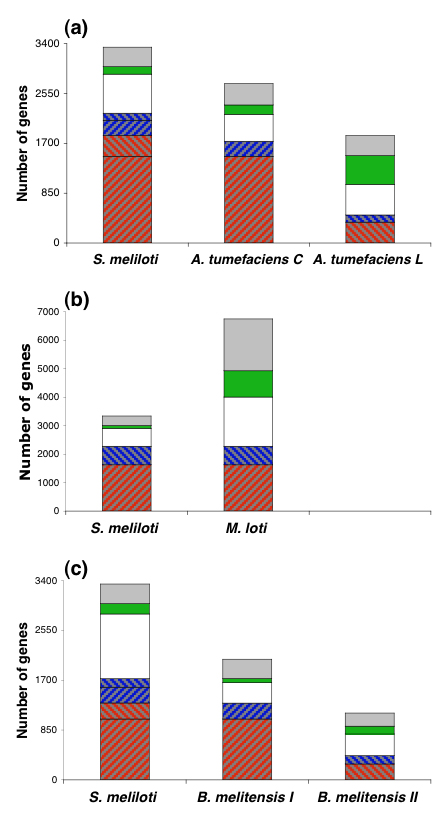

Supplement: Additional File 1 — Schematic representation of the Rhizobiales chromosomes in comparison with S. meliloti, according to the gene classification of predicted orthologs and homologs. Panels: (a), S. meliloti-A. tumefaciens comparison. (b), S. meliloti-M. loti comparison. (c), S. meliloti-B. melitensis comparison. Red striped bars, syntenic genes with the organism in comparison. Blue striped bars, non-syntenic genes with the organism in comparison. White bars, homologs with other Rhizobiales chromosomes (for S. meliloti, compare with Fig. 1, white fraction). Green bars, homologs in plasmids. Gray bars, species-specific genes. In panels a and c, the S. meliloti chromosome shows syntenic and non-syntenic genes with both replicons of the organisms under comparison. Red striped bars, syntenic genes, lower fraction: with (a) At-C and (c) Bm-I chromosomes; upper fraction: with (a) At-L and (c) Bm-II chromosomes. Blue striped bars, non-syntenic genes, lower fraction: with (a) At-C and (c) Bm-I chromosomes; upper fraction: with (a) At-L and (c) Bm-II chromosomes. [file 1471-2148-5-55-S1.jpeg]

## Slide 1
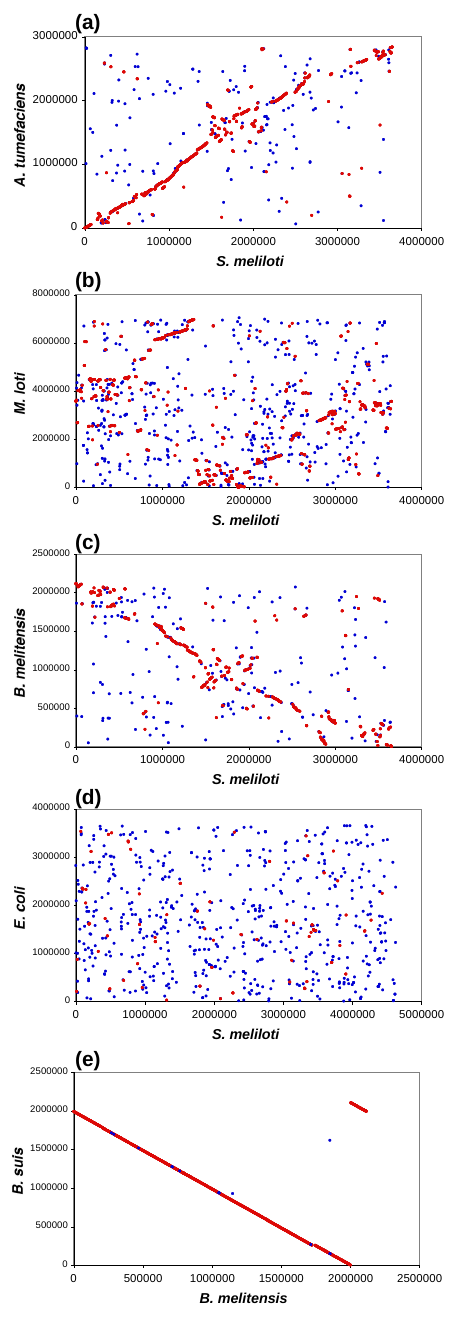

(a)
(b)
(c)
(d)
(e)

Supplement: Additional File 4 — Synteny of Rhizobiales and Enterobacteriales. Panels: (a), S. meliloti-A. tumefaciens circular chromosomes comparison. (b), S. meliloti-M. loti comparison. (c), S. meliloti-B. melitensis chromosome I comparison. (d), S. meliloti-E. coli comparison. (e), B. suis-B. melitensis chromosomes I comparison. Red dots, syntenic genes. Blue dots, non-syntenic genes. Scales in bp. [file 1471-2148-5-55-S4.ppt]

## Slide 1
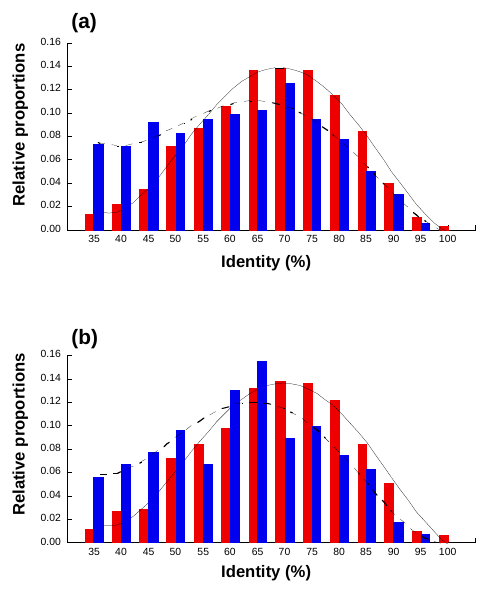

(a)
Relative proportions
Identity (%)
(b)
Relative proportions
Identity (%)

Supplement: Additional File 6 — Sequence identity distribution of chromosomal translated orthologs. Panels: (a), syntenic and non-syntenic products from the S. meliloti-M. loti comparison. (b), syntenic and non-syntenic products from the S. meliloti-B. melitensis (chromosomes I and II) comparison. Y-axis, relative proportions. Red bars, syntenic genes. Blue bars, non-syntenic genes. Y-axis, relative proportions. [file 1471-2148-5-55-S6.ppt]

## Slide 1
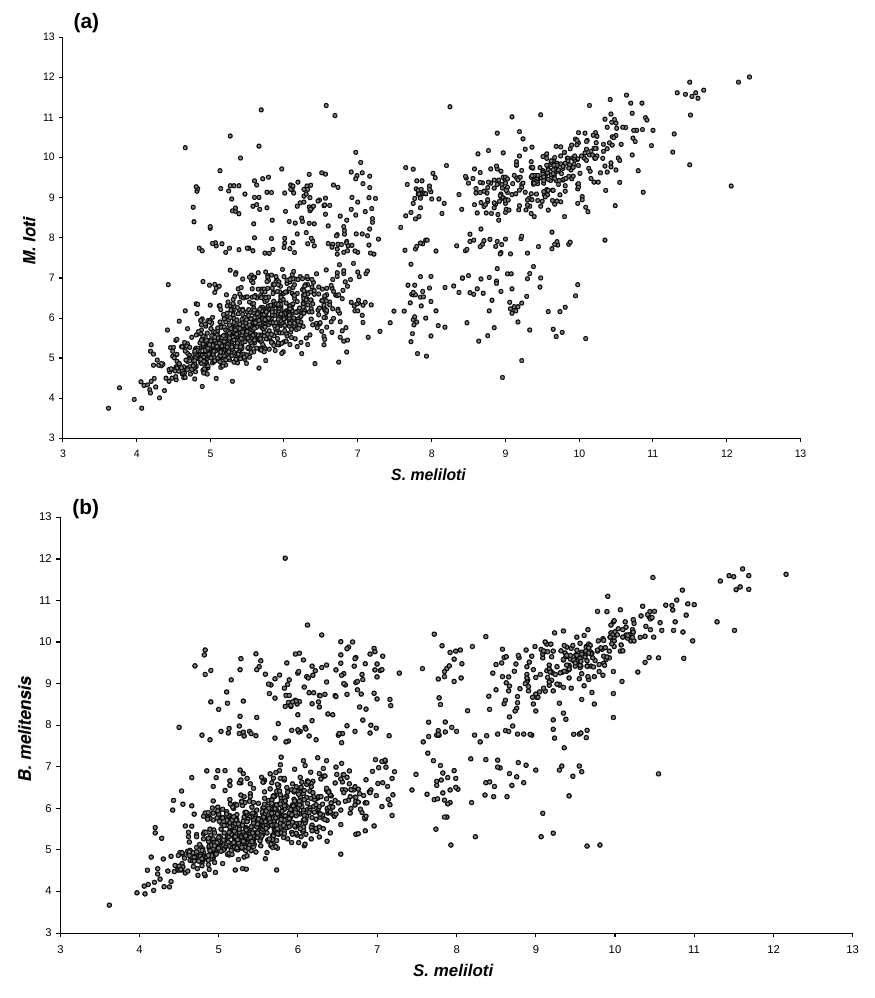

(a)
(b)

Supplement: Additional File 8 — Theoretical isoelectric points (pI) of syntenic products. Panels: (a), S. meliloti-M. loti comparison. (b), S. meliloti-B. melitensis (chromosomes I and II) comparison. Dots represent translated products. Scales in pH units. [file 1471-2148-5-55-S8.ppt]

## Slide 1
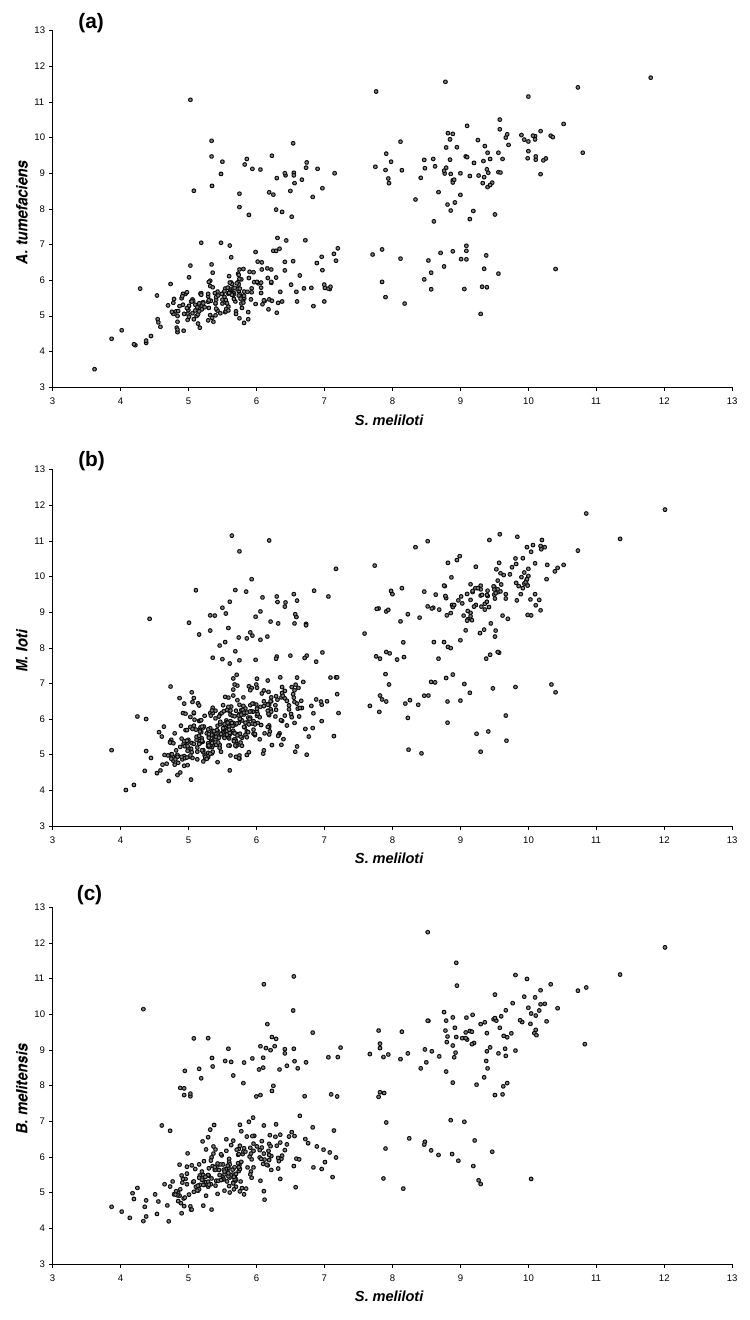

(a)
(b)
(c)

Supplement: Additional File 10 — Theoretical isoelectric points (pI) of nonsyntenic products. Panels: (a), S. meliloti-A. tumefaciens (both chromosomes) comparison. (b), S. meliloti-M. loti comparison. (c), S. meliloti-B. melitensis (both chromosomes) comparison. Dots represent translated products. Scales in pH units. [file 1471-2148-5-55-S10.ppt]

## Slide 1
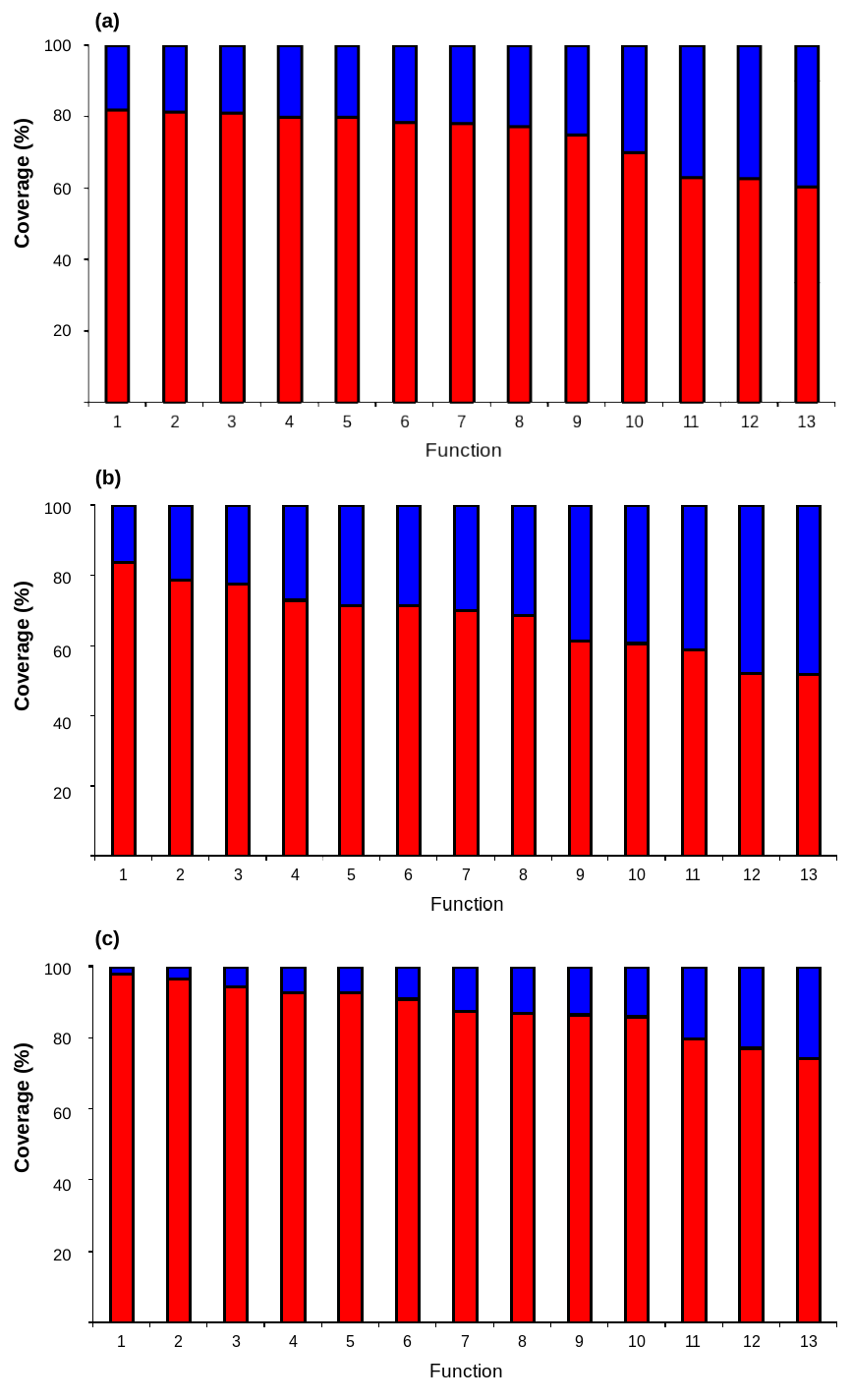

(a)
100
80
Coverage (%)
60
40
20
(b)
100
80
Coverage (%)
60
40
20
(c)
100
80
Coverage (%)
60
40
20

Supplement: Additional File 13 — Coverage of functional classes with syntenic and non-syntenic genes. Panels: (a), S. meliloti-M. loti comparison. Classes: 1) Translation, 2) Transcription, 3) Purine, pyrimidine, nucleoside and nucleotide metabolism, 4) Cellular processes, 5) Energy metabolism, 6) Cell envelope, 7) Fatty acid and phospholipid metabolism, 8) Biosynthesis of cofactors, prosthetic groups and carriers, 9) Transport and ATP binding proteins, 10) Amino acid metabolism, 11) DNA metabolism, 12) Regulatory functions, 13) Central intermediary metabolism. (b), S. meliloti-B. melitensis comparison. Classes: 1) Transcription, 2) Translation, 3) Cellular processes, 4) Biosynthesis of cofactors, prosthetic groups and carriers, 5) Cell envelope, 6) Energy metabolism, 7) Fatty acid and phospholipid metabolism, 8) Purine, pyrimidine, nucleoside and nucleotide metabolism, 9) Amino acid metabolism, 10) Transport and ATP binding proteins, 11) DNA metabolism, 12) Regulatory functions, 13) Central intermediary metabolism. (c), E. coli-E. carotovora comparison. Classes: 1) Biosynthesis of cofactors, prosthetic groups and carriers, 2) Purine, pyrimidine, nucleoside and nucleotide metabolism, 3) Translation, 4) Fatty acid and phospholipid metabolism, 5) Transcription, 6) Cellular processes, 7) DNA metabolism, 8) Energy metabolism, 9) Amino acid metabolism. 10) Cell envelope, 11) Regulatory functions, 12) Transport and ATP binding proteins, 13) Central intermediary metabolism. Note that order of classes is different to that in Fig. 8. Red bars, syntenic genes. Blue bars, non-syntenic genes. [file 1471-2148-5-55-S13.ppt]

## Slide 1
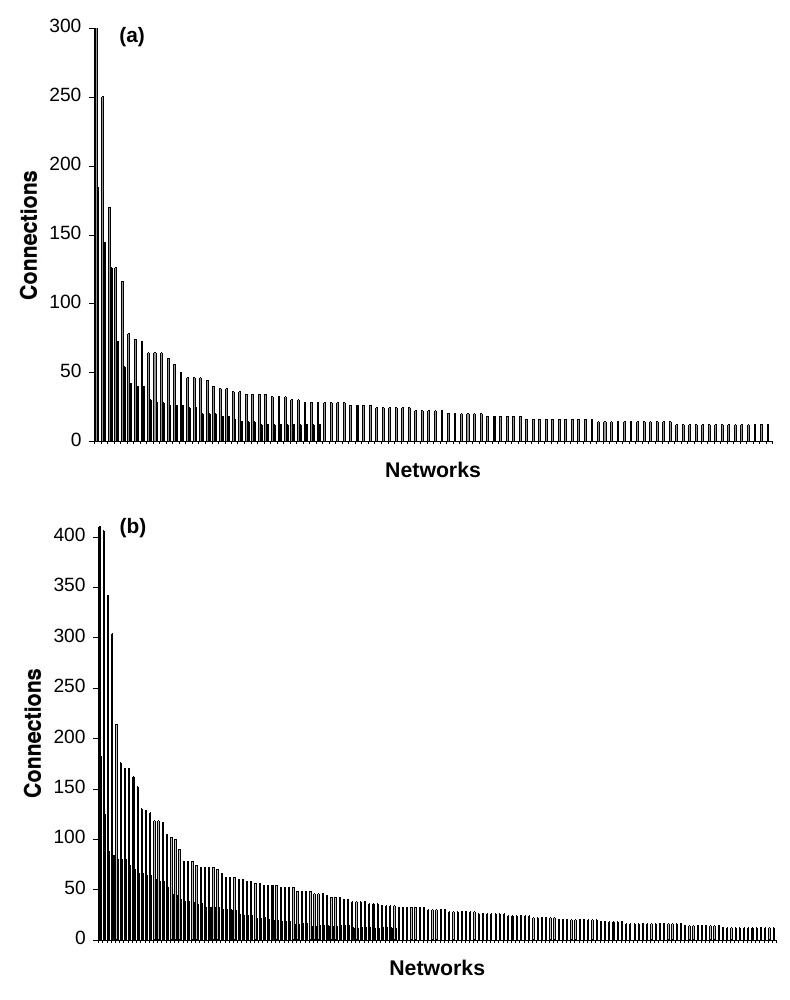

(a)
(b)

Supplement: Additional File 14 — Connectivity values from networks formed by microsyntenic and non-conserved regions in (a) S. meliloti (in comparison with A. tumefaciens) and (b) E. coli (in comparison with E. carotovora). Y-axis, connections per network. First syntenic networks, with 1060 (S. meliloti) and 810 (E. coli) connections, were omitted for clarity. Arranged in decrecent connectivity order. Gray bars, microsyntenic regions. Black bars, non-conserved regions. Successive networks, with connectivity values lower than 6, were omitted. [file 1471-2148-5-55-S14.ppt]
