# Supplementary material for: Evolutionary, structural and functional relationships revealed by comparative analysis of syntenic genes in Rhizobiales
Source: BMC Evol Biol. 2005 Oct 17;5:55. doi: 10.1186/1471-2148-5-55 (PMC1276791; doi:10.1186/1471-2148-5-55)
Supplement: Additional File 7 — Sequence alignments and data from the alignments of proteins from the arginine biosynthetic pathway in Rhizobiales and Enterobacteriales. Panels: (a), ArgC in Rhizobiales. Identical residues for aech position are marked with yellow. Least abundant residues for a given position are denoted with an specific color for each of the species: dark blue, differences in R. palustris; green, differences in B. melitensis; red, differences in M. loti; gray, differences in A. tumefaciens; violet, S. meliloti. (b), ArgC in Rhizobiales and Enterobacteriales. Identical residues for aech position are marked with yellow. Least abundant residues for a given position are denoted with an specific color for each of the species: Rhizobiales, same code of panel (a). Enterobacteriales: brown, Buchnera; pink, E. carotovora; blue, S. typhimurium. E. coli and S. flexneri, none. (c), data of the identity (*) and similarity (:*) in residues and in percentage (bold) of the alignments of the proteins in Rhizobiales and Enterobacteriales. [file 1471-2148-5-55-S7.pdf]

(a)

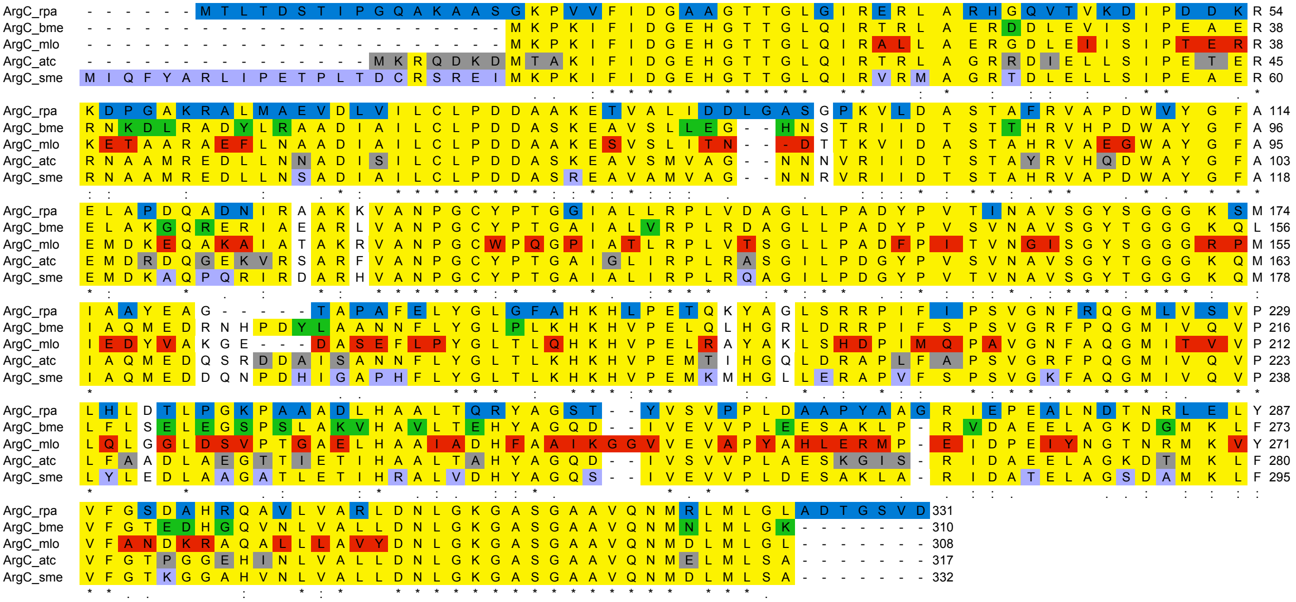

	pI
Rpa	6.1
Bme	5.9
Mlo	5.4
Atu	5.5
Sm	6.0

Identical residues	111
All residues different	9
Changed residues in seq1	113
Changed residues in seq2	33
Changed residues in seq3	83
Changed residues in seq4	41
Changed residues in seq5	48

(b)

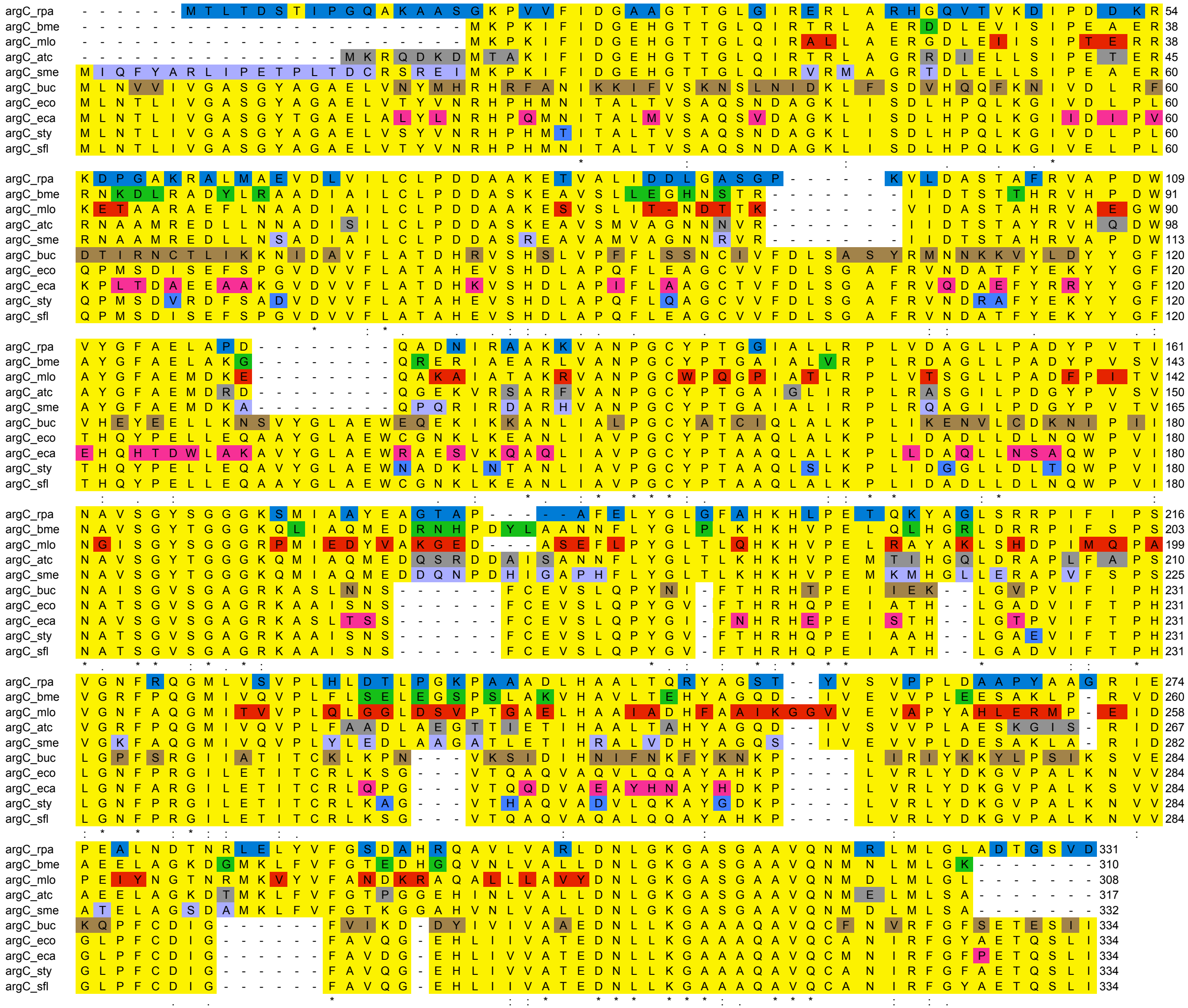

	pI
Rpa	6.1
Bme	5.9
Mlo	5.4
Atu	5.5
Sm	6.0
Buc	9.2
Eco	5.6
Eca	5.9
Sty	5.7
Sfl	5.6

Identical residues	38
All residues different	0
Changed residues in seq1	99
Changed residues in seq2	35
Changed residues in seq3	79
Changed residues in seq4	42
Changed residues in seq5	54
Changed residues in seq6	113
Changed residues in seq7	0
Changed residues in seq8	49
Changed residues in seq9	16
Changed residues in seq10	0

(c)

*	ArgB		ArgC		ArgD		ArgF		ArgG		ArgH	
**Rhizob.**	173	**62.0**	111	**33.9**	101	**24.7**	163	**50.6**	102	**23.4**	260	**56.0**
**Enterobac.**	157	**56.3**	171	**52.3**	211	**51.6**	180	**55.9**	101	**23.2**	265	**57.1**
**both**	56	**20.1**	38	**11.6**	67	**16.4**	74	**23.0**	68	**15.6**	137	**29.5**

*:	ArgB		ArgC		ArgD		ArgF		ArgG		ArgH	
**Rhizob.**	226	**81.0**	170	**52.0**	191	**46.7**	211	**65.5**	191	**43.9**	326	**70.3**
**Enterobac.**	208	**74.6**	236	**72.2**	304	**74.3**	244	**75.8**	209	**48.0**	361	**77.8**
**both**	108	**38.7**	76	**23.2**	143	**35.0**	130	**40.4**	141	**32.4**	238	**51.3**
