# Supplementary material for: Evolutionary, structural and functional relationships revealed by comparative analysis of syntenic genes in Rhizobiales
Source: BMC Evol Biol. 2005 Oct 17;5:55. doi: 10.1186/1471-2148-5-55 (PMC1276791; doi:10.1186/1471-2148-5-55)
Supplement: Additional File 9 — Summary of proteins with differential pI's from comparisons with chromosomes of S. meliloti, A. tumefaciens (circular) and M. loti. [file 1471-2148-5-55-S9.doc]

| Summary of proteins with differential pI´s from comparisons with chromosomes of *S. meliloti, A. tumefaciens* (circular) and *M. loti*. | | | |
| --- | --- | --- | --- |
| *Sm* | *At* | *Ml* | No. of proteins |
|  |  | A | 615 |
|  | A | N | 24 |
|  |  | B | 48 |
|  |  | A | 11 |
| A | N | N | 2 |
|  |  | B | 4 |
|  |  | A | 34 |
|  | B | N | 5 |
|  |  | B | 23 |
|  |  | A | 17 |
|  | A | N | 1 |
|  |  | B | 7 |
|  |  | A | 1 |
| N | N | N | 1 |
|  |  | B | 3 |
|  |  | A | 4 |
|  | B | N | 3 |
|  |  | B | 10 |
|  |  | A | 22 |
|  | A | N | 5 |
|  |  | B | 13 |
|  |  | A | 2 |
| B | N | N | 1 |
|  |  | B | 10 |
|  |  | A | 13 |
|  | B | N | 8 |
|  |  | B | 197 |
| With similar pI (A-A, or N-N, or B-B) | | 813 (75.0%) |  |
| With low variability (A to N, N to B) | | 91 ( 8.4) |  |
| With high variability (A to B) | | 153 (14.1) |  |
| Variability in all range (A to N- to B) | | 27 ( 2.5) |  |
|  | |  |  |
| A, Acid. N, Neutral. B, Basic. | |  |  |
