# Supplementary material for: Evolutionary, structural and functional relationships revealed by comparative analysis of syntenic genes in Rhizobiales
Source: BMC Evol Biol. 2005 Oct 17;5:55. doi: 10.1186/1471-2148-5-55 (PMC1276791; doi:10.1186/1471-2148-5-55)
Supplement: Additional File 11 — Functional categories of syntenic products of the membranal prediction of S. meliloti- A. tumefaciens (circular chromosome) comparison. [file 1471-2148-5-55-S11.doc]

Functional categories of syntenic products of the membranal prediction in *S. meliloti-A. tumefaciens* (circular chromosome) comparison.

|  |  |  |
| --- | --- | --- |
| Syntenic products | 1480 |  |
|  |  |  |
|  | **With membranal segments** | **Without membranal segments** |
|  |  |  |
|  | 790 (53%) | 690 (47%) |
|  |  |  |
| With functional assignment | 481 (61%) | 441 (64%) |
|  |  |  |
| Function unknown | 309 (39%) | 249 (36%) |
|  |  |  |
| **Metabolism** | 186 (39%) | 181 (41%) |
|  |  |  |
| Energy obtention | 52 | 20 |
| Cofactor synthesis | 32 | 27 |
| Amino acid metabolism | 29 | 25 |
| Intermediary metabolism | 26 | 46 |
| General function | 20 | 28 |
| Carbohydrate metablism | 11 | 7 |
| Fatty acids metabolism | 9 | 13 |
| Nucleotide metabolism | 7 | 15 |
|  |  |  |
| **Cellular Processes** | 277 (57%) | 158 (36%) |
|  |  |  |
| Transport | 127 | 27 |
| Posttranslational  modification | 53 | 30 |
|  |  |
| Signal transduction | 29 | 41 |
| Cellular surface | 29 | 18 |
| Chemotaxis | 24 | 17 |
| Cell division | 8 | 6 |
| Cell protection | 4 | 3 |
| Adaptation | 3 | 7 |
| Chaperoning | 0 | 9 |
|  |  |  |
| **Informational** | 18 (4%) | 102 (24%) |
|  |  |  |
| Transcription | 0 | 14 |
| Replication/Recombination | 1 | 23 |
| Translation | 17 | 65 |
